# Adolescent Somatic Symptoms Under Psychosocial Adversity: Differential Links of Risk and Protective Factors to Physical and Psychological Burden in Türkiye

**DOI:** 10.3390/children13080985

**Published:** 2026-07-24

**Authors:** Derya Azim, Sevde Betül Kara, Muhammed Emre Güvey, Ecenur Aydemir, Sümeyra Gündem, Salim Yılmaz

**Affiliations:** 1 Department of Physiotherapy and Rehabilitation, Faculty of Health Sciences, Bandırma Onyedi Eylül University, Balıkesir 10200, Türkiye; dazim@bandirma.edu.tr; 2 Department of Physiotherapy and Rehabilitation, Faculty of Health Sciences, Istanbul Arel University, İstanbul 34010, Türkiye; betulyigit@arel.edu.tr; 3 Department of Healthcare Management, Faculty of Health Sciences, Acibadem Mehmet Ali Aydinlar University, İstanbul 34752, Türkiye; emre.guvey@acibadem.edu.tr (M.E.G.); ecenur.aydemir@acibadem.edu.tr (E.A.); 4 Department of Healthcare Management, Faculty of Health Sciences, Istanbul Arel University, İstanbul 34010, Türkiye; sumeyraefe@arel.edu.tr; 5 Department of Healthcare Management, Graduate School of Health Sciences, Acibadem Mehmet Ali Aydinlar University, İstanbul 34752, Türkiye

**Keywords:** adolescents, somatic symptoms, internalizing symptoms, psychosocial adversity, risk and protective factors, resilience, peer victimization, parental support, Türkiye

## Abstract

**Highlights:**

**What are the main findings?**

- Each one-standard-deviation increase in peer victimization was associated with 84% higher odds of greater psychological symptom burden, whereas each one-standard-deviation increase in parental support was associated with 12% lower odds; both associations were significantly stronger for psychological than for physical symptoms.
- Girls had 2.35 times the odds of greater physical symptom burden, and adolescents with a chronic illness had 61% higher odds. Body mass index, household income strain, and housing problems showed no independent associations with either symptom dimension.

**What is the implication of the main finding?**

- Recurrent symptoms in adolescents may give clinicians and school health services reason to ask about peer victimization, school alienation, parental support, and chronic illness rather than limiting assessment to the presenting somatic complaint.
- The eight-item symptom checklist may be useful for monitoring psychosocially patterned adolescent symptom burden at the population level, but the modest predictability observed here indicates that the measured risk and protective factors should not be used for individual-level screening or prediction.

**Abstract:**

**Background/Objectives:** Adolescent somatic symptoms such as headache, irritability, and sleep difficulties are common and closely tied to adolescent mental health, yet they are typically studied as a single dimension and as individual complaints detached from the psychosocial adversity surrounding the adolescent. Drawing on the nationally representative 2022 Türkiye Child Survey (*n* = 3523, ages 13–17), we examined whether these symptoms are patterned by the psychosocial risk and support surrounding the adolescent. **Methods:** Measurement models, multiple correspondence analysis, survey-weighted ordinal regression, a mixed graphical model, and machine-learning algorithms were applied in sequence. **Results:** Somatic symptoms were organized around a dominant general factor, alongside closely correlated physical and psychological dimensions that showed differential external associations. The three relational microsystems formed a coherent psychosocial adversity gradient along which somatic burden increased. The two dimensions were linked to different factors: peer victimization, a risk factor, and parental support, a protective factor, were associated primarily with psychological burden (odds ratios per standard deviation 1.84 and 0.88), whereas female sex and chronic illness were linked more strongly to physical burden (2.35 and 1.61); body mass index, income strain, and housing problems showed no independent associations. Regression, network, and machine-learning analyses converged on this dissociation while indicating modest individual-level predictability. **Conclusions:** Adolescent somatic symptoms are thus systematically patterned by psychosocial risk and support at the population level, an association that may inform monitoring and psychosocially informed assessment rather than individual prediction, and that requires longitudinal work to interpret directionally.

## 1. Introduction

Recurrent physical and emotional complaints such as headache, stomachache, backache, dizziness, low mood, irritability, and disturbed sleep are among the most common health problems reported by adolescents worldwide [1,2]. In international surveys, a substantial share of adolescents report at least one such symptom every week, and the prevalence has risen over the past two decades, particularly among girls [1]. Although these symptoms rarely indicate identifiable organic disease, they are far from trivial. They tend to co-occur, increase with age, and are more frequent among girls [1,3], and they are associated with impaired daily functioning, school absence, and reduced quality of life. Their high prevalence and these associated burdens place adolescent somatic complaints among the health outcomes shaped by the social determinants of adolescent health [4], making them a question of population health rather than of individual clinical care alone. Throughout, we follow the structure of the instrument that records them. The Health Behaviour in School-aged Children Symptom Checklist registers eight complaints that fall into two content clusters. A physical cluster comprises headache, stomachache, backache and dizziness, and a psychological cluster comprises feeling low, irritability, nervousness and difficulty sleeping. We refer to the second as psychological somatic burden, and we use that term consistently to mean those four items and nothing else. It is a component of a checklist of psychosomatic complaints, not a measure of mental disorder, and it is not offered as a proxy for one. Its content does overlap with what is commonly called internalizing distress. It is worth noting at the outset that the boundary is not sharp in the literature either, since internalizing problems are themselves defined inconsistently across instruments, with somatic complaints included in some and excluded from others [5].

How these symptoms are understood, however, often remains narrow. They are frequently approached either as residual medical puzzles to be excluded once organic causes are ruled out, or as purely psychological phenomena located within the individual child. Both framings tend to detach the symptom from the social environments in which the adolescent lives. The biopsychosocial model long ago argued that physical complaints arise at the intersection of biological, psychological, and social processes rather than within any one of them [6,7], yet in practice somatic symptoms are still commonly studied one determinant at a time, with family, school, peer, and socioeconomic influences examined in isolation rather than as interacting risk and protective influences.

A systems perspective offers a more coherent framing. Bronfenbrenner’s ecological theory situates the developing adolescent within nested systems, from the immediate microsystems of family, school, and peer group to the broader socioeconomic context, each interacting to shape health and development [8]. Read through this lens, a somatic symptom is not merely an internal event but a point at which psychosocial adversity in the adolescent’s environment may become legible in the body. Ecological and systems perspectives, which examine how interconnected conditions produce health outcomes [9], are consistent with the broader literature on psychosocial risk and protective factors that shape adolescent mental health. What an ecological framework does not by itself supply, however, is an account of translation. It identifies the systems that surround the adolescent, but it does not say how a condition in one of them comes to be felt as a headache, or as a night without sleep. That step requires a psychological mechanism, and in its absence the claim that adversity becomes legible in the body is a metaphor rather than a hypothesis.

Two mechanisms supply that step, and they do not act on the same symptoms. The first is appraisal. Stress arises not from demands as such, but from demands appraised as exceeding the resources available to meet them, and adolescence concentrates such demands, among them academic evaluation, peer comparison and shifting family relations, at a point when the resources for meeting them are still forming. Across a decade of Norwegian survey data, stress in the school domain remained associated with subjective health complaints after adjustment for sex, age and the other stress domains, and the association with school attendance was stronger in girls. The authors attribute this to how stress is appraised and managed, rather than to exposure alone [10]. The resources that buffer appraisal are, at this age, largely relational, and the family supplies a substantial share of them. A meta-analysis of 49 studies and 24,524 young people found that a child’s capacity to regulate emotion mediated the association between several family factors, among them secure attachment, psychological control, family conflict and unsupportive emotion socialization, and internalizing symptoms [11]. When regulation is overwhelmed, what appears is not, in the first instance, pain. It is low mood, irritability, tension and disturbed sleep, precisely the four complaints that constitute the psychological dimension of the checklist.

The second mechanism is perception. A bodily sensation does not become a symptom automatically. It becomes one through an inferential process in which prior expectations, attention, and affect determine whether an interoceptive signal is registered as noise or as pain. The correspondence between physiological state and reported symptom is therefore highly variable, both between individuals and within the same individual over time, and depends as much on the person and the context as on the input itself [12]. This route can generate bodily complaints without any change in underlying physiology, and it has a developmental driver of its own during exactly the years examined here. In early childhood, boys and girls differ neither in the prevalence of primary pain nor reliably in experimental pain sensitivity, but from around the age of twelve girls become more sensitive to painful stimuli and their rates of primary pain, pain that is the problem itself rather than a consequence of tissue damage, begin to rise sharply [13].

What follows is not that adversity necessarily produces somatic symptoms, but that its associations may differ across the two symptom dimensions. If the appraisal route runs through affect, the factors that load on it, among them victimization, alienation and the absence of parental support, should attach preferentially to the psychological cluster. If the perceptual route runs through the body, factors that act on bodily state and on pain processing, such as female sex and chronic illness, should attach preferentially to the physical one. A single undifferentiated symptom score cannot express such a pattern, and this is the theoretical reason to expect two dimensions rather than one [10,13].

This has a direct consequence for the disciplines to which these symptoms are actually brought. Of the eight complaints, it is the physical ones, recurrent pain above all, that bring an adolescent into a clinic, and the mechanism just described implies that the pain arriving there may have little to do with the tissue being examined. Physiotherapists and other health professionals are often among the first to see these complaints [14,15]. Contemporary physiotherapy has itself moved toward a biopsychosocial understanding of pain, recognizing that musculoskeletal and other physical symptoms in young people are seldom explained by tissue pathology alone and are shaped by the psychosocial context in which they occur [14]. If a symptom is a point of entry into a system rather than a lesion, then the question at that point of entry is not only where it hurts but what surrounds the adolescent who hurts. The implication does not stop at physiotherapy. It reaches school health services, which tend to see the absence rather than the symptom, and adolescent mental health services, which tend to see the distress but not the body. An adolescent may be seen in turn by all three, with none of them positioned to see the pattern that the symptoms form.

Three gaps motivate the present study. Individual microsystems have each been linked to adolescent somatic complaints, with peer victimization [16,17] and the family environment [18,19] among the most studied, yet these influences are rarely examined together within a single framework that treats them as co-occurring layers of one system. The symptoms themselves are also commonly treated as a single, undifferentiated dimension, and cross-national measurement work has generally found the most widely used checklist to be largely unidimensional [20]. As a result, the distinction between physical and psychological symptom burden, and whether different systems relate to each in different ways, has received little attention. Compounding both issues, the evidence remains concentrated in high-income Western settings, while nationally representative data from middle-income countries such as Türkiye are scarce, even though the social conditions surrounding adolescents differ markedly across contexts.

The present study addresses these gaps using nationally representative data from the 2022 Türkiye Child Survey. Working within the ecological framework and the two mechanisms set out above, we map adolescent somatic symptom burden onto the systems that surround it, namely family support, the school experience, peer victimization, lifestyle and health behaviors, and socioeconomic circumstances. Three expectations follow from that account. First, that somatic symptoms in this population are better represented by two correlated dimensions than by one. Second, and centrally, that the two dimensions attach differently to the system, in the direction the mechanisms predict, with the relational microsystems of peer victimization, school alienation, and parental support more closely associated with psychological burden and with female sex and chronic illness more closely associated with physical burden. Third, that the direct associations of socioeconomic conditions with somatic burden should be weak once the relational microsystems are accounted for, a pattern compatible with, though not demonstrative of, an indirect pathway through the proximal environments in which those conditions are lived.

This is a secondary analysis of an existing survey and was not preregistered. The measurement structure was established before the mechanism outlined above was articulated, and the differential pattern was first observed in the regression. The measurement stage and the descriptive mapping are therefore reported as exploratory. The formal test of differential attachment, in which both dimensions are regressed simultaneously on the full predictor set and their paths constrained to equality, is reported as a formal within-sample test of a pattern already described, and not as independent confirmation of it. Establishing that would require external replication, ideally preregistered. The mechanism generates predictions that reach beyond that pattern, including the asymmetry of sex and chronic illness, and these are tested.

## 2. Materials and Methods

The analysis proceeds through several methods, each answering a different question about the same system, and none of them substituting for the others. Confirmatory factor analysis assesses whether the symptoms have the two-dimensional structure predicted by the mechanism. Multiple correspondence analysis asks whether the three relational microsystems co-occur as a single gradient of adversity rather than acting as independent exposures. This describes whether categories drawn from the three microsystems co-occur along a common gradient rather than varying independently of one another. Latent profile analysis asks whether the lifestyle and socioeconomic indicators form reproducible subgroups rather than acting as independent continuous exposures; its failure to recover a defensible profile structure is the reason those indicators are carried forward separately. Survey-weighted ordinal regression asks which layers of the system are independently associated with each dimension. A latent-variable model asks whether the two dimensions differ, formally, in how they attach to that system. A mixed graphical model asks what remains once every variable is conditioned on every other, and so which associations are direct within the measured set and which run through other nodes. Machine learning asks how much of an individual adolescent’s burden the measured system can account for at all.

The final two methods support description rather than causal inference. A network estimated from cross-sectional data does not identify causal structure, since an undirected edge is compatible with an entire equivalence class of causal structures, and recovering the network does not select among them [21]. It serves to check whether the associations found by regression persist after conditioning on the whole system. The machine-learning importances likewise describe the fitted model rather than the world.

### 2.1. Data Source and Study Population

This study is a secondary analysis of microdata from the 2022 Türkiye Child Survey (TCA 2022), a nationally representative, cross-sectional survey conducted by the Turkish Statistical Institute (TURKSTAT) in collaboration with UNICEF Türkiye [22]. The survey was carried out under a bilateral protocol between TURKSTAT and the Ministry of Family and Social Services, and a memorandum of understanding between TURKSTAT and UNICEF Türkiye, and its field application was completed throughout Türkiye between 10 October and 16 December 2022. The microdata set analyzed here was obtained from the official TURKSTAT microdata service on 3 March 2026 for the purposes of the present study, in accordance with the data confidentiality provisions of Türkiye Statistics Law No. 5429.

The survey used a multistage cluster sampling design intended to produce estimates at the national level. In the first stage, 901 clusters, each consisting of approximately 100 household addresses with at least one child aged 0 to 17 years, were selected with probability proportional to size; in the second stage, 10 household addresses were selected from each cluster by systematic random sampling. Interviews were completed in 7716 households. Information on 14,705 children aged 0 to 17 years was obtained from mothers or primary caregivers, and a separate self-report questionnaire was administered directly to 4072 adolescents aged 13 to 17 years. The present study draws on this adolescent self-report module, which covers life satisfaction, school life, somatic and psychological symptoms, parental relationships, peer relationships, and child participation.

TURKSTAT provided final person-level sampling weights for the 13- to 17-year module (FAKTOR_FERT), constructed through design weighting, nonresponse adjustment, integrative calibration, trimming, and an overall inflation factor, so that weighted estimates represent the national population of adolescents in this age range. Stratum and primary sampling unit identifiers were not released with the microdata.

### 2.2. Measures

#### 2.2.1. Somatic Symptoms

Somatic symptom burden was measured with the eight-item HBSC Symptom Checklist (HBSC-SCL), a self-report instrument developed within the WHO-collaborative Health Behavior in School-aged Children study, in which Türkiye has previously participated, to capture recurrent psychosomatic complaints in adolescence [2]. The instrument has demonstrated good validity and cross-national measurement properties in large multinational adolescent samples [2,20]. Adolescents reported how often during the previous six months they had experienced four physical complaints (headache, stomachache, backache, and dizziness) and four psychological complaints (feeling low, irritability, feeling nervous, and difficulties falling asleep). In the source data, the frequency response options were not ordered, so each item was recoded onto a five-point frequency scale (0 = rarely or never, 1 = about once a month, 2 = about once a week, 3 = several times a week, 4 = about every day), with higher scores indicating greater symptom burden. These response options follow the original survey instrument and were treated as a monotonically increasing frequency scale. Because confirmatory factor analysis supported two correlated dimensions rather than a single one (Supplementary Material, Section S3), we computed a physical and a psychological subscale, each as the mean of its four items, together with a total score across all eight items (range 0 to 4).

Each somatic outcome was analyzed as a three-level ordered variable. The total somatic score, used in the descriptive and correspondence analyses, was divided at its sample tertiles into low, moderate, and high burden. The physical and psychological subscales were instead categorized at fixed cut-points, because their distributions were strongly zero-inflated (31.8% of adolescents reported no physical symptom and 27.7% no psychological symptom) and could not be split into tertiles: adolescents scoring zero formed the low category, and the remaining adolescents were divided at a subscale cut-point (0.75 for the physical and 1.0 for the psychological dimension, each approximately the median of the non-zero scores) into moderate and high burden.

#### 2.2.2. School Alienation

The adolescent’s experience of the school environment was assessed using items from the survey’s seven-item school experience module, which is based on the Psychological Sense of School Membership scale [23], a scale that has been adapted and validated for use with Turkish students [24]. The full module combined positively worded items on belonging and being liked with negatively worded items on exclusion, awkwardness, loneliness, and examination anxiety, rated on a five-point agreement scale (1 = strongly disagree to 5 = strongly agree). Rather than treating the module as a single belonging score, we evaluated its factor structure in the present sample and retained the three items that formed a coherent and reliable factor, describing feeling excluded or left out, feeling awkward and out of place, and feeling lonely at school. The four remaining items, concerning positive belonging and examination anxiety, did not load with these and were not retained; the full item-selection procedure is reported in the Supplementary Material, Section S2. The three retained items were scored so that higher values indicate greater school alienation.

#### 2.2.3. Peer Victimization

Exposure to bullying was measured with six items asking how often during the previous 12 months the adolescent had experienced each of six forms of victimization: being deliberately excluded, being made fun of, being threatened, having belongings taken or destroyed, being pushed around, and having rumors spread about them. In the source data, the response options included intermediate categories such as several times a year and several times a month, which were recoded onto a five-point frequency scale (0 = rarely or never to 4 = every day) so that higher values reflect more frequent exposure. These items are part of the standardized HBSC bullying module used across HBSC member countries. A mean exposure score was computed across the six items, which formed a strong single factor in confirmatory analysis (Cronbach’s α = 0.93). The module measured victimization only; it did not include items on perpetration of bullying.

#### 2.2.4. Parental Support

The perceived quality of parental support was measured with items rated on a five-point agreement scale, drawn from a module conceptually aligned with the family subscale of the Multidimensional Scale of Perceived Social Support [25], an instrument adapted and validated for Turkish samples [26]. The module contained eight items, six describing supportive behaviors (parents providing help, allowing valued activities, showing care, understanding worries, encouraging independent decisions, and providing comfort) and two describing parental control (treating the adolescent like a baby and trying to control everything they do). Confirmatory factor analysis showed that the two control items did not load on the support dimension and represented a distinct construct, so they were excluded; the six supportive items were retained and averaged into a parental support score (range 1 to 5; Cronbach’s α = 0.918), with higher scores indicating greater perceived support. The item-selection procedure is reported in the Supplementary Material, Section S2.

#### 2.2.5. Body Mass Index

Body mass index (BMI) was computed from caregiver-reported height and weight as weight in kilograms divided by the square of height in meters. Implausible values (BMI < 8 or >60) were treated as missing. Because BMI varies systematically with age and sex during adolescence, it was standardized to a within-sample z-score within each age-by-sex cell (using cells with at least ten observations), so that the resulting variable expresses each adolescent’s relative weight status for their age and sex, with higher values indicating higher relative weight.

#### 2.2.6. Diet

Dietary intake was measured with food-frequency items, recoded so that higher values indicate more frequent consumption (0 = never to 6 = every day). A healthy diet score was computed as the mean of fruit and vegetable consumption, and an unhealthy diet score as the mean of sweets, salty snacks, and sugar-sweetened soft drink consumption (Cronbach’s α = 0.73 for the unhealthy diet score), so that a higher healthy diet score reflects more frequent intake of fruit and vegetables and a higher unhealthy diet score more frequent intake of energy-dense, nutrient-poor items. The two scores were treated as distinct dimensions rather than combined, since healthy and unhealthy intake may co-occur.

#### 2.2.7. Physical Activity, Chronic Illness, and Context

Physical activity was measured as the number of days in the previous seven on which the adolescent had been physically active for at least 60 min (range: 0 to 7), and participation in organized sport as a binary indicator of taking part in an amateur or professional sporting activity in the previous 12 months. The presence of a chronic illness was recorded in the household health module, completed by the mother or primary caregiver, as a binary indicator of any ongoing illness or an illness experienced in the previous six months, with a follow-up item naming the condition; it was reported rather than physician-verified. Household income strain was measured on a five-point scale indexing the difficulty the household experienced in meeting its basic needs (1 = very easily to 5 = with great difficulty), and housing problems as the mean of five dwelling-condition indicators (damp walls, decay in window frames or floors, a leaking roof, excessive noise, and insufficient daylight), with higher values indicating poorer conditions. Age in completed years and sex were drawn from the household roster.

### 2.3. Missing Data and Analytic Sample

Of the 4072 adolescents, we restricted all analyses to those with complete data on the full set of analytic variables (*n* = 3523; 86.5%). Missingness was concentrated in the items underlying the school constructs, each with missing rates between 11 and 13 percent, reflecting a scoring rule under which “not applicable” responses were treated as missing; the remaining variables were either complete or near-complete. Little’s test rejected the assumption that data were missing completely at random (χ^2^ = 468.23, df = 180, *p* < 0.001). Comparison of included and excluded participants showed that excluded adolescents were markedly older, with 60.5% aged 16 or 17 compared with 35.7% of those included, and were more often female and reported greater household income strain. The two groups did not differ in somatic symptom burden, which suggests no selection on the observed outcome. This age gradient follows from the scoring rule because the school items did not apply to adolescents who had left formal education; thus, the excluded group is likely to consist disproportionately of adolescents no longer attending school. The analytic sample carries a weighted total of 5.57 million adolescents, compared with 6.46 million for the full survey file, and therefore represents approximately 86% of the weighted national population aged 13 to 17. Because the analytic chain propagates derived scores across stages and integrates methods that do not accommodate full-information estimation, we adopted a single complete-case sample throughout to maintain an identical case base; sensitivity analyses under full-information maximum likelihood yielded substantively identical measurement results. Full details of the missing data analysis are provided in the Supplementary Material, Section S1, Table S1.

As a sensitivity analysis, both ordinal regression models were repeated on the full survey file (*N* = 4072) after multiple imputation. Missing values were imputed at the item level by predictive mean matching, with 30 imputations and five iterations, and with all analytic variables and the sampling weight included in the imputation model [27]. Subscale scores, outcome categories, and standardized predictors were rebuilt within each imputed dataset; the survey-weighted models were refitted; and the estimates were combined by Rubin’s rules [28]. No association changed direction or crossed the conventional threshold of significance. Multiple imputation assumes that the data are missing at random given the observed variables, an assumption that cannot be verified from the data themselves. Because the missing values here are almost entirely confined to the school items, which were not applicable to adolescents outside formal schooling, that assumption is precisely the one in doubt, and the imputation is therefore a statistical sensitivity check rather than a remedy for the underlying limitation (Supplementary Material, Section S1).

### 2.4. Analytic Strategy

Throughout, we use the terms risk factor and protective factor in their conventional epidemiological sense, to denote correlates associated with a higher or lower level of an outcome, without implying temporal precedence or causation, neither of which the present cross-sectional design can establish. A measurement stage established the structure of the somatic symptom outcome and the psychosocial indices, an exploratory stage examined how the psychosocial microsystems and the lifestyle and socioeconomic indicators were configured, and an explanatory stage related these to somatic burden. Confirmatory factor analysis was applied to all reflective multi-item self-report scales, namely the somatic symptom outcome, school alienation, parental support, and peer victimization, to establish their dimensionality and reliability before use. Formative or composite indices that are defined by their components rather than by a reflective latent construct, such as the diet and housing-problem scores, were not subjected to confirmatory factor analysis and were used as derived scores. The measurement, geometric, and person-centered models (confirmatory factor analysis, multiple correspondence analysis, and latent profile analysis, respectively) are concerned with recovering structure rather than estimating population quantities, and the available survey microdata did not include stratum or cluster identifiers. Because stratum and primary sampling unit identifiers were not released with the microdata, the design could be specified with sampling weights alone. Any clustering in the sample is therefore unaccounted for, and the standard errors reported here are, to that extent, likely to be underestimated [29]. The robustness of each association to a range of plausible design effects was examined by inflating the standard errors accordingly; the core associations remained significant at a design effect of 2, whereas four of the smaller associations, parental support, chronic illness and physical activity with psychological burden, and school alienation with physical burden, did not survive a design effect of 3 (Supplementary Material, Section S5, Table S14). The same inflation was applied to the Wald tests of the path differences in the latent model, by multiplying the standard error of each coefficient difference by the square root of the assumed design effect; all four core differences remained significant at an assumed design effect of 2, and the omnibus tests at a design effect of 3 (Supplementary Material, Section S6, Table S16). The measurement, geometric, and person-centered models were therefore estimated on unweighted data, under the assumption that the sampling was not strongly informative for these structural relationships. All population-level quantities, including descriptive prevalences and the final associations between the lifestyle and socioeconomic indicators, psychosocial position, and somatic burden, were estimated with the sampling weight applied through a survey design object (survey package [30]), so that prevalence estimates and regression coefficients generalize to the population represented by the analytic sample. Because missingness was concentrated in the school items, that population is best understood as adolescents aged 13 to 17 who were attending school, rather than as all adolescents in this age range (Section 2.3). This hybrid approach mirrors established practice for complex survey data, in which design structure is used for inference on population quantities while structural models are fit to the unweighted sample [31].

The two somatic dimensions were each modeled as a three-level ordered outcome (low, moderate, high) using survey-weighted ordinal logistic regression under the proportional odds assumption. Because both somatic scores were right-skewed with a substantial floor (zero) component, each dimension was categorized as follows: a low level comprising adolescents reporting no symptoms on that dimension, and moderate and high levels defined by thresholds that divided the remaining, nonzero distribution into approximately equal-sized groups (physical: 0, >0 to 0.75, >0.75; psychological: 0, >0 to 1.0, >1.0). The composite somatic symptom category used in the descriptive table and the correspondence analysis was derived analogously from the total symptom score, partitioned into low, moderate, and high groups of approximately equal size. All cut-points were applied to the unweighted scores; survey weights were used in estimation, not in defining the categories. For an outcome $Y_{i}$ with categories j = 1, 2, 3 the model specifies.

$$logit\left[\mathrm{Pr}\left(Y_{i}\leq j\right)\right]=\alpha_{j}-\sum_{k=1}^{K}\beta_{k}x_{ik}\;,\;j=1,2$$

where $\alpha_{j}$ are the ordered category thresholds, $\beta_{k}$ the regression coefficients for the K predictors $x_{ik}$, and $exp(\beta_{k})$ the odds ratio for a one-unit change in the predictor k, assumed constant across thresholds. The two dimensions were fitted as parallel models with an identical predictor set, with all continuous predictors standardized so that the resulting odds ratios are comparable across predictors, and the proportional odds assumption was assessed by comparing the predictor coefficients across the two cumulative binary thresholds defined by each outcome (≥moderate and = high), following the graphical approach of Harrell [32]. Departures from proportionality were small for the physical outcome (mean absolute difference in log-odds 0.06) and for the psychological outcome (0.10), with the exception of peer victimization, whose association with psychological burden was somewhat stronger at the lower threshold. The proportional odds model was retained because the assumption held for every other predictor and because the direction and significance of each association were consistent across thresholds (Supplementary Material, Section S5).

All confirmatory measurement models were estimated with the WLSMV estimator using the delta parameterization, treating the five-category indicators as ordinal. Model fit was judged against the comparative fit index and Tucker–Lewis index, with values of 0.90 or above indicating acceptable fit, and the root mean square error of approximation and standardized root mean square residual, with values of 0.08 or below indicating acceptable fit; competing models were compared by the scaled chi-square difference test. Measurement adequacy was assessed through standardized factor loadings, internal consistency (ordinal coefficient alpha and McDonald’s omega), and composite reliability, and convergent validity was evaluated using the average variance extracted [33]. For the two correlated somatic factors, discriminant validity was assessed using the Fornell–Larcker criterion and the heterotrait–monotrait ratio of correlations [34]. A bifactor model was fitted as an additional candidate structure for the somatic items, in which every item loads on a general somatic factor and on an orthogonal cluster-specific factor. Because a bifactor model is more flexible than its competitors and tends to fit better irrespective of the underlying structure, it was evaluated through its loadings and through the bifactor indices, namely explained common variance, percentage of uncontaminated correlations, item-level explained common variance, omega hierarchical, omega hierarchical subscale, and construct replicability, rather than through model fit [35,36,37].

To test whether the two dimensions were differentially associated with the system, rather than merely comparing two separately fitted models, we specified a multiple-indicator multiple-cause model in which both latent somatic factors were regressed simultaneously on the full predictor set [38]. The model was estimated with the WLSMV estimator, delta parameterization, and sampling weights, with the latent factors scaled to unit variance and all continuous predictors standardized. Because both regressions are estimated within a single model, the covariance between any pair of coefficients is available, and the equality of the two paths for a given predictor was tested by a Wald test of the corresponding constraint, applied to each predictor individually and jointly to predefined blocks. This model was specified after the regression results had been obtained. It is therefore a formal test of a pattern first observed descriptively within the same sample, not a preregistered hypothesis and not independent confirmation.

Because the ordinal specification requires the continuous subscale scores to be categorized, the associations were re-estimated under alternative specifications of the outcome: a four-level outcome retaining zero as its own category and dividing the non-zero scores into tertiles; a two-part hurdle model separating the presence of any symptom from its severity among those reporting symptoms; a linear model of the standardized continuous score; and a repetition of the primary model with latent factor scores replacing the mean-score psychosocial predictors. The pattern of association was reproduced under every specification (Supplementary Material, Section S5).

#### 2.4.1. Network Analysis

To complement the regression, which relates each predictor to somatic burden one variable at a time, we estimated a mixed graphical model (MGM) to recover the conditional dependence structure of the system as a whole [39]. Each variable is a node, and each edge is a regularized partial association between two nodes conditional on all others. Because the variables were of mixed type, continuous nodes were modeled as conditionally Gaussian and binary nodes as categorical, so that no variable was forced onto an inconsistent measurement scale. Edge weights were estimated by nodewise $l_{1}$-regularized (LASSO) regression, in which each node $X_{s}$ was regressed on all remaining nodes $X_{\backslash s}$ by minimizing

$${\hat{\beta }}_{s}=arg\underset{\beta_{s}}{min}\left\{\frac{1}{n}\sum_{i=1}^{n}l\left(x_{is},\;\beta_{s0}+\sum_{r\neq s}\beta_{sr}\;x_{ir}\right)+\lambda_{s}\sum_{r\neq s}\left|\beta_{sr}\right|\right\}$$

where $l\left(\cdot \right)$ is the negative log-likelihood appropriate to the type of node s (Gaussian or categorical) and $\lambda_{s}$ the regularization parameter selected by the extended Bayesian information criterion. An edge was retained only when both nodewise regressions identified it (AND rule). For each node, we computed its strength centrality, the sum of the absolute weights of its edges, and its predictability, the variance explained by its neighbors. Network accuracy and stability were assessed by nonparametric and case-dropping bootstraps (1000 resamples each), with centrality stability summarized by the correlation-stability coefficient [40]. As with the other structural models, the network was estimated on the unweighted sample (Supplementary Material, Section S7).

#### 2.4.2. Machine-Learning Validation

As a complementary analysis, we used machine learning to assess the relative contribution of each predictor to the two somatic dimensions. Each three-category outcome was modeled with two tree-based ensembles able to capture nonlinearity and interactions without imposing a functional form, random forest and extreme gradient boosting, alongside elastic-net penalized regression as an interpretable linear baseline against which any gain from the ensembles could be judged. Models were trained on a stratified 70% partition and evaluated on the held-out 30% test set, with all hyperparameters tuned by 10-fold cross-validation within the training partition under a scheme shared across algorithms; discrimination was summarized by the multiclass area under the curve. Predictor importance was quantified by permutation importance and by Shapley additive explanations (SHAP). Full details are given in the Supplementary Material, Section S8.

All analyses were conducted in R version 4.5.2 (R Foundation for Statistical Computing, Vienna, Austria); the full set of packages used at each stage of the analysis, together with their versions and purpose, is documented in the Supplementary Material, Section S9, Table S21 [41,42,43,44,45,46,47,48,49,50,51,52,53,54,55,56,57,58,59,60,61,62,63,64,65,66,67,68].

## 3. Results

The analytic sample comprised 3523 adolescents aged 13 to 17 years, weighted to represent the population represented by the analytic sample, which consists predominantly of adolescents attending school. Sample demographic, lifestyle, and socioeconomic characteristics are summarized in Table 1.

Just over half were male, and the weighted somatic symptom category distribution was 40.6% low, 29.8% moderate, and 29.6% high. Most adolescents lived in households reporting some degree of income strain and reported infrequent physical activity, with a median of zero days per week meeting the recommended threshold. Organized sport participation was limited (21.4%), and chronic illness was reported by roughly one in ten adolescents (Table 1).

We first established the measurement structure of the constructs that anchor the analysis: the somatic symptom outcome, the experience of school alienation, perceived parental support, and peer victimization. Each was measured with multi-item self-report scales adapted from established instruments, and we evaluated the dimensionality and reliability of each before relating them to the broader socioecological context. Confirmatory factor models were estimated with the WLSMV estimator, which is appropriate for the ordinal, positively skewed response distributions observed here, and convergent and discriminant validity were assessed alongside internal consistency (Table 2).

For the somatic symptom scale, a one-factor model treating all complaints as a single dimension fit poorly (RMSEA = 0.132), whereas a two-factor model separating physical from psychological complaints fit well across all indices (CFI = 0.987, TLI = 0.981, RMSEA = 0.073, SRMR = 0.046), and the scaled chi-square difference test confirmed its superiority (Δχ^2^ = 428.67, Δdf = 1, *p* < 0.001). Standardized loadings were strong throughout, ranging from 0.668 to 0.757 on the physical factor and from 0.576 to 0.893 on the psychological factor. The two factors were closely related but distinguishable. The heterotrait–monotrait ratio (0.79) remained below the 0.85 threshold, whereas the Fornell–Larcker criterion was not satisfied for the physical factor, whose square root of the average variance extracted (0.708) fell below the interfactor correlation (0.733). Distinguishability therefore rests not on a low correlation, which is substantial, but on three converging lines of evidence: the two-factor advantage replicated in two independent random halves of the sample (Δχ^2^ = 196.01 and 233.53, both *p* < 0.001); the interfactor correlation was stable across those halves (0.728 and 0.739); and the two dimensions had significantly different external correlates, the constraint that all predictor paths are equal across dimensions being decisively rejected (Table 3). Sharing a substantial common symptom core is thus compatible with attaching differently to the surrounding system.

A bifactor model, fitted as a third candidate structure, sharpened this picture rather than altering it (Supplementary Material, Section S2, Table S7). It fitted better than the one- and two-factor models, as a more flexible bifactor model almost always does, so its interpretation rested on the loadings and the bifactor indices. A strong general factor ran through all eight items, accounting for most of the reliable variance in the total score (omega hierarchical 0.785; explained common variance 0.724). The physical and psychological subscale scores, considered on their own, therefore carried little reliable variance unique to their cluster (omega hierarchical subscale 0.183 and 0.210, respectively), and two items, difficulty sleeping and, to a lesser extent, feeling dizzy, carried almost no cluster-specific information. The two-dimensional structure thus resides at the latent level and in the external relations of the dimensions rather than in the raw subscale scores, which are better read as indicators of a single somatic burden. The bifactor MIMIC model provided an additional sensitivity analysis of this differential pattern. After accounting for the general factor, the specific-factor paths were not uniformly equal (χ^2^(13) = 69.04, *p* < 0.001), with female sex loading twice as strongly on the physical specific factor as on the psychological one (0.800 against 0.418, difference *p* < 0.001). These residual-factor coefficients are nonetheless interpreted cautiously, because the specific factors showed limited reliability and construct replicability.

The school alienation, parental support, and peer victimization factors each loaded strongly on a single dimension, with loadings of 0.699 to 0.838, 0.727 to 0.883, and 0.752 to 0.878, respectively. Both the parental support and school alienation factors required item selection. Two parental support items concerning parental control did not load on the support dimension and were removed, and two residual covariances were freed between items with overlapping content; items that did not load on the intended dimension were likewise removed from the school alienation module (Table S3). These decisions were guided by the data rather than specified in advance, and we report them as exploratory. Their stability was examined by repeating the measurement analyses in two random halves of the sample: both freed residual covariances ranked among the three largest modification indices in the full sample and in each half, with a positive expected parameter change throughout, as expected of items sharing content rather than of sampling noise (Supplementary Material, Section S2, Table S6). Because the school alienation factor retains three items and is just-identified when specified alone, its global fit was evaluated within a five-factor model containing all multi-item constructs, which fit the data well (CFI = 0.988, RMSEA = 0.034; Table S5). Composite reliability was high across all constructs (0.80 to 0.93), and the average variance extracted supported convergent validity, exceeding the conventional 0.50 threshold for every factor except the physical somatic factor, whose value reached 0.50 and whose adequacy is further supported by its high composite reliability (Table S4). Internal consistency was good to excellent throughout (α = 0.791 to 0.930). Taken together, the checklist is dominated by a general symptom factor with residual physical and psychological clustering. The correlated two-factor representation was retained for analyses of differential external associations, with the two dimensions interpreted as closely related rather than independent. School alienation, parental support, and peer victimization each form coherent and reliable constructs carried forward into the subsequent analyses (Table 2).

Across the weighted sample, somatic symptom burden was modest on average but widely dispersed. The general somatic score averaged 0.80 (SD 0.75; median 0.62 [0, 3.88]) on the 0 to 4 frequency scale, with the psychological subscale (mean 0.95, SD 0.96; median 0.75 [0, 4]) somewhat higher than the physical subscale (mean 0.64, SD 0.73; median 0.50 [0, 4]). Adolescents reported moderate school alienation (mean 2.02, SD 0.63; median 2.00 [1, 5]) and high perceived parental support (mean 4.00, SD 0.58; median 4.00 [1, 5]) on their respective five-point scales. Peer victimization was low and strongly right-skewed, with most adolescents reporting little or no exposure (mean 0.16, SD 0.43; median 0.00 [0, 4]).

**Figure 1:**
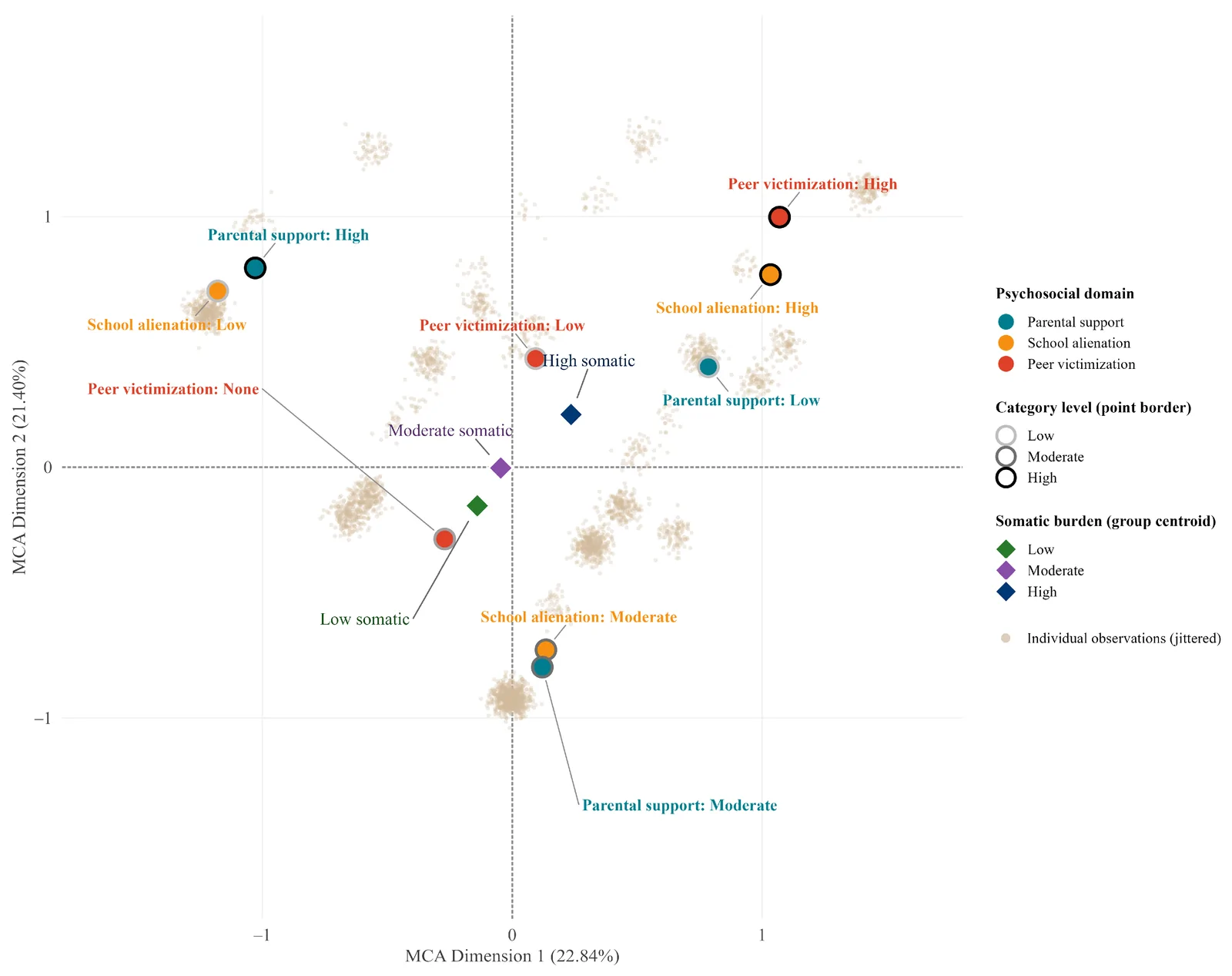
Multiple correspondence analysis of peer victimization, school alienation, and parental support. Category points show each level of the three domains in the space of the first two MCA dimensions, with point border thickness denoting the ordered category level (none, low, or high for peer victimization; low, moderate, or high for school alienation and parental support). Diamonds mark the centroids of the three somatic-burden groups, and faint points show individual adolescents (jittered). Dimension percentages refer to the share of inertia explained. Because the analysis takes three categorical variables as input, adolescents with identical category profiles share identical coordinates; the visible spread therefore reflects the jitter rather than variation in the data.

Having established the measurement properties of the three psychosocial constructs, we examined how they jointly structure the adolescent’s relational context. Because peer victimization, school alienation, and parental support each capture a distinct microsystem and were only modestly intercorrelated (absolute Spearman correlations 0.10 to 0.28), we used multiple correspondence analysis as an exploratory tool to map how their categories are configured in a shared space, rather than to derive a single composite (Figure 1).

The first dimension, accounting for 22.8% of the raw inertia (62.9% after Benzécri adjustment [69]), described a coherent gradient of psychosocial adversity: the supportive poles of all three domains (low school alienation, high parental support, and no peer victimization) clustered at one end, and their adverse poles at the other. Adolescents with greater somatic burden were positioned further along this adverse end, with the low, moderate, and high somatic-burden centroids ordered monotonically along the first dimension. Each domain showed a significant ordinal trend with somatic-burden category (peer victimization Z = 17.33, school alienation Z = 10.11, parental support Z = −4.64, all *p* < 0.001; Supplementary Material, Section S3, Tables S8–S10). At the same time, the three domains loaded unequally on the first dimension (squared correlations 0.26 for peer victimization, 0.48 for parental support, and 0.63 for school alienation), and the dimension captured only part of their joint variation, indicating that each microsystem carried information not reducible to a single adversity axis. We therefore treated the three constructs as separate predictors in the subsequent models, while retaining the first MCA dimension as a composite adversity score for a sensitivity analysis (Figure 1). The multiple correspondence analysis is descriptive. It summarizes how the categories of the three microsystems co-occur in this sample and does not itself test the gradient it displays. The raw inertia of the first dimension is accordingly modest, as is characteristic of multiple correspondence analysis, in which the coding of the indicator matrix deflates the raw figure and motivates the Benzécri adjustment reported above. The claim that this dimension corresponds to accumulating adversity rests not on the inertia but on the monotone ordering of somatic burden along it and on the ordinal trends reported here, which are tested separately in the models that follow.

We next examined whether the lifestyle and socioeconomic indicators (body mass index, diet, physical activity, income strain, and housing problems) formed distinct latent profiles. The indicators were almost entirely uncorrelated (all |r| < 0.11 except income strain with housing problems, r = 0.29; Figure S1), and latent profile models (one to six profiles, compared across four covariance parameterizations and evaluated by the Bayesian information criterion, entropy, and the bootstrap likelihood-ratio test) did not yield a defensible profile structure, with the Bayesian information criterion declining monotonically and entropy remaining low across solutions (Supplementary Material, Section S4, Table S11). We therefore entered these indicators as separate predictors in the subsequent models, alongside organized sport participation and chronic illness, rather than as a derived profile variable.

**Figure 2:**
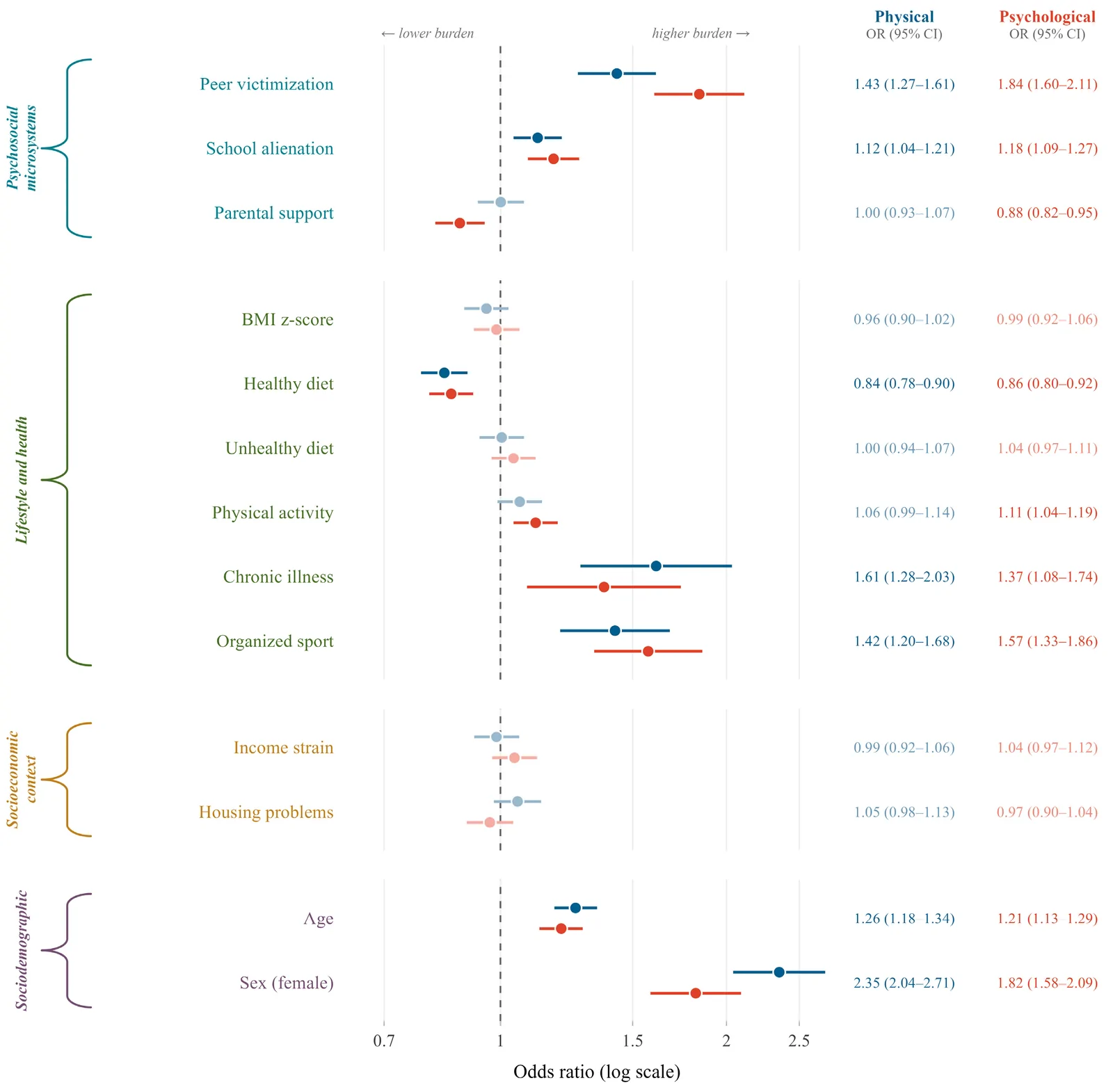
Adjusted associations of system-level factors with physical and psychological somatic burden. Points are odds ratios from two parallel survey-weighted ordinal regressions, one per somatic dimension (blue, physical somatic burden; red, psychological somatic burden), with 95% confidence intervals on a log scale; values are listed at right. The dashed vertical line marks an odds ratio of one; odds ratios above one indicate greater burden, and faded points mark intervals that include one. Full results are reported in Supplementary Material, Table S12.

To identify which system layers were most closely tied to somatic burden, we fitted two parallel survey-weighted ordinal regressions, one for each somatic dimension, entering the three psychosocial microsystems, the lifestyle and socioeconomic indicators, and sociodemographic covariates simultaneously (Figure 2; full coefficients in Supplementary Material, Section S5).

The two dimensions were associated with partly distinct sets of factors. Predictors were placed on a common scale by standardizing all continuous variables, so that each odds ratio refers to a one-standard-deviation increase and magnitudes are directly comparable across predictors. Peer victimization was the strongest psychosocial correlate of both, but its association was larger for the psychological dimension than for the physical one (odds ratio 1.84 per standard deviation, 95% CI 1.60 to 2.11 versus 1.43, 95% CI 1.27 to 1.61). School alienation was associated with both dimensions to a similar, more modest degree (1.18, 95% CI 1.09 to 1.27 versus 1.12, 95% CI 1.04 to 1.21), whereas parental support was associated with lower psychological burden (0.88, 95% CI 0.82 to 0.95) but was unrelated to physical burden (1.00, 95% CI 0.93 to 1.07). The sociodemographic and bodily factors showed the complementary pattern: being female and having a chronic illness were each associated with higher burden on both dimensions but more strongly on the physical one (sex, odds ratio 2.35 versus 1.82; chronic illness, 1.61 versus 1.37), and older age with higher burden on both. A healthier diet was associated with lower burden on both dimensions, and organized sport participation with higher burden on both; more frequent physical activity was associated with higher psychological burden only, while body mass index, unhealthy diet, household income strain, and housing problems were not independently associated with either dimension. The psychosocial associations were unchanged when the three microsystems were combined into a single composite adversity axis, and generalized additive models indicated linear associations for both age and body mass index, with no U-shaped relationship between body mass index and somatic burden (Supplementary Material, Section S5).

The comparison in Figure 2 is informative, but it is not a test. The two dimensions were modeled separately, so no covariance between a pair of coefficients is available and the precision of their difference cannot be recovered; the coefficients also inherit measurement error in the observed subscale scores, which need not attenuate them equally, and they depend on the categorization of the outcome. We therefore re-estimated the associations within a single multiple-indicator multiple-cause model, in which the physical and psychological somatic factors, each defined by its four ordinal items, were regressed simultaneously on all 13 predictors, with sampling weights applied, the latent factors scaled to unit variance, and all continuous predictors standardized, so that coefficients are directly comparable across the two dimensions. Because both regressions are estimated within one model, the difference between any pair of paths can be tested by a Wald test of the corresponding equality constraint. This removes the categorization of the outcome, corrects for measurement error, and converts the comparison between the two dimensions from a juxtaposition of point estimates into a formal test (Table 3).

The constraint that all 13 paths are equal across the two dimensions was decisively rejected (χ^2^(13) = 83.74, *p* < 0.001), as were the constraints applied separately to the three psychosocial paths (χ^2^(3) = 31.86, *p* < 0.001) and to the two biological and demographic paths (χ^2^(2) = 31.34, *p* < 0.001). Five path differences reached significance in this model, but four are robust, and one is not. The four that were reproduced under every sensitivity specification, and that we therefore interpret, are as follows. Peer victimization was more strongly associated with psychological burden (Δβ = −0.062, *p* = 0.003), and the association of parental support was confined to that dimension (physical β = −0.026, *p* = 0.268; psychological β = −0.103, *p* < 0.001; Δβ = 0.076, *p* < 0.001). In the opposite direction, female sex (Δβ = 0.218, *p* < 0.001) and chronic illness (Δβ = 0.193, *p* = 0.003) were more strongly associated with physical burden. The fifth difference, for housing problems, reached *p* = 0.042 in the primary model, but neither of its individual paths is significant (physical *p* = 0.086; psychological *p* = 0.815), and it was not reproduced in the other specifications. We do not interpret it. Tested jointly, these four constraints were rejected at χ^2^(4) = 54.14, *p* < 0.001. The differences for school alienation (*p* = 0.057) and organized sport (*p* = 0.150) did not reach significance in this model, and although both did so under some alternative specifications, neither is treated as robust (Supplementary Material, Section S6, Table S15).

Two features of this result qualify it. The two dimensions remain closely related after conditioning on all 13 predictors: the correlation between their latent residuals was 0.686, and the predictors explained an almost identical share of variance in each (R^2^ = 0.179 and 0.179). Their separability therefore does not rest on their being weakly correlated, which they are not, but on their having significantly different external correlates. The pattern was also reproduced when the outcomes were modeled as continuous scores, under an alternative categorization, under a two-part hurdle specification, and after multiple imputation of the full survey file, and the four core differences remained significant under an assumed design effect of 2 (Supplementary Material, Section S1, Table S2; Section S5, Table S13; Section S6, Table S16).

**Figure 3:**
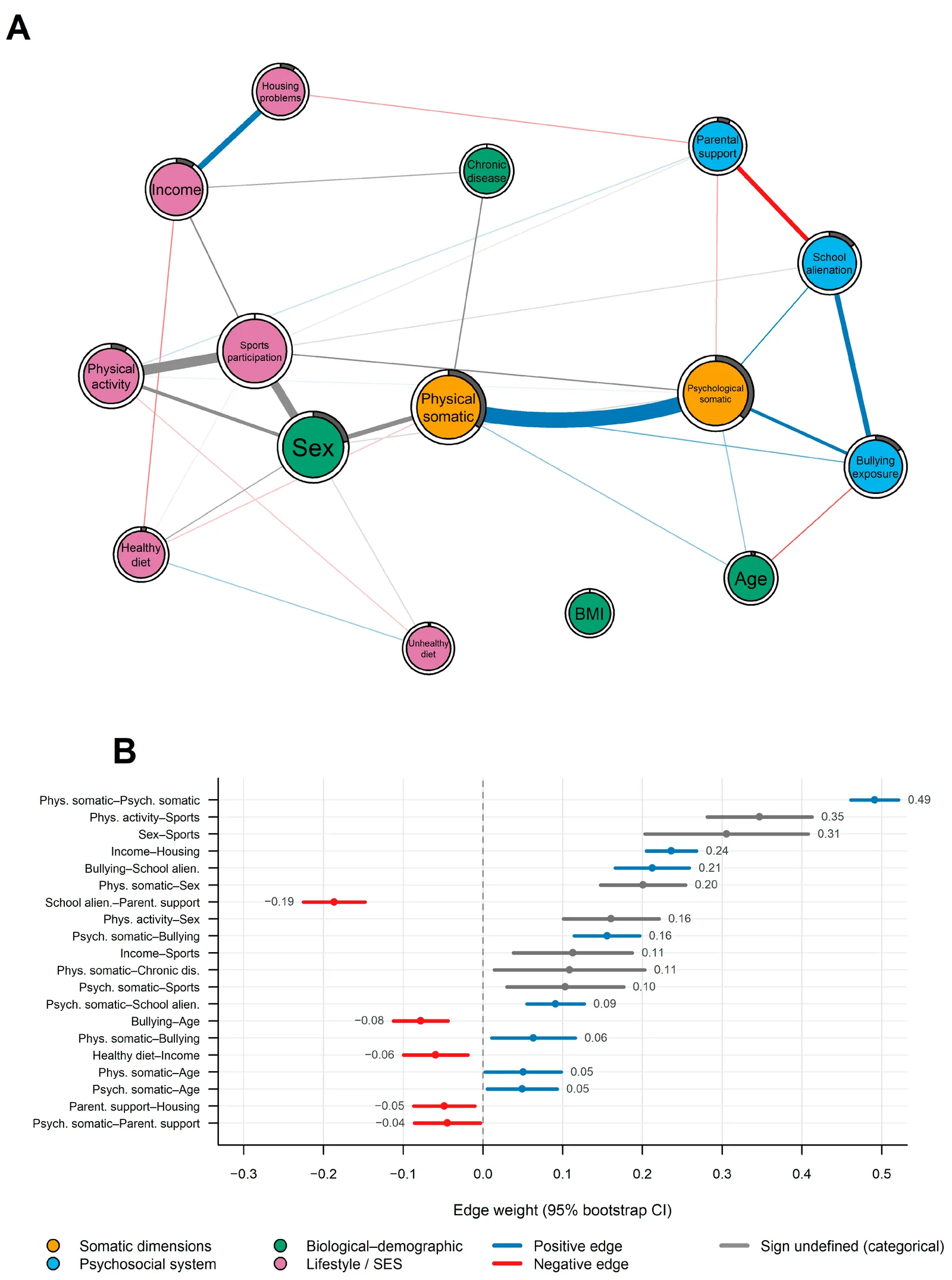
Mixed graphical model of the somatic dimensions and their system-level correlates. Nodes are colored by system (somatic, psychosocial, biological–demographic, and lifestyle or socioeconomic); edge thickness is proportional to the absolute partial association, with blue and red denoting positive and negative associations and gray denoting edges to categorical nodes, whose sign is undefined at the edge level. Node size reflects strength centrality, and the ring around each node shows its predictability: the black segment of the ring marks the proportion of the node's variance explained by its neighbors, and the white remainder the unexplained proportion. BMI z-score retained no edges: no association with any other node survived regularization. Its isolation in the graph is a result, not an artifact of the layout. Panel (**A**) shows the network; panel (**B**) shows the bootstrap-validated edges with their 95% confidence intervals.

The regression and latent models relate each factor to somatic burden one at a time but do not show how the two somatic dimensions are situated within the wider web of variables, nor whether they connect to different parts of that web. Whereas the regression asks how strongly each predictor relates to somatic burden, a network representation asks a complementary question: how the somatic dimensions are embedded among their correlates, which variables occupy central positions in the system, and whether physical and psychological burden attach to different regions of it. To recover this system-level structure, we estimated a mixed graphical model in which each variable is a node and each edge represents a regularized partial association conditional on all others, accommodating the continuous, binary, and count variables together (Figure 3).

The network was sparse, retaining 23 of 105 possible edges, of which 20 were supported by 95% bootstrap confidence intervals that excluded zero, and its centrality ordering was highly stable under case-dropping resampling (correlation-stability coefficient = 0.75). Full numerical detail is reported in Supplementary Material, Section S7, Table S17.

The network and the regression address different questions, because every edge in the network is estimated conditional on all remaining variables, including the other somatic dimension. The strongest edge in the entire network, by a wide margin, connected the two somatic dimensions to each other (partial association 0.49, 95% CI 0.46 to 0.52), confirming a substantial shared somatic core that persists after conditioning on every other variable. Beyond this core, the two dimensions attached to distinct regions of the system. Physical burden connected to the biological and demographic nodes: the edge to sex (0.20, 95% CI 0.15 to 0.25) indicated that being female was associated with higher physical burden, and the edge to chronic illness (0.11, 95% CI 0.01 to 0.20) indicated that adolescents with a chronic illness carried higher physical burden, each conditional on all other variables. Psychological burden connected instead to the psychosocial nodes: greater peer victimization (0.16, 95% CI 0.11 to 0.20) and greater school alienation (0.09, 95% CI 0.06 to 0.13) were both associated with higher psychological burden, whereas greater parental support was associated with lower psychological burden (−0.04, 95% CI −0.09 to 0.00). The psychosocial nodes also cohered among themselves, with peer victimization and school alienation directly connected (0.21) and parental support linked negatively to school alienation (−0.19), recovering the internal structure of the relational microsystem. The lifestyle and socioeconomic nodes connected largely among themselves, in expected and mechanical ways (physical activity with organized sport, 0.35; income strain with housing problems, 0.24). Organized sport nonetheless retained a direct edge to psychological burden (0.10), whereas its association with physical burden did not survive conditioning; in the conditional models reported in Table S18, the odds ratio for the physical association of sport fell from 1.42 to 1.18 (complete-case nodewise coefficient 0.02, *p* = 0.48; 0.01, *p* = 0.71 after multiple imputation), while its psychological association persisted. Both somatic dimensions ranked among the most central nodes in the network, with psychological and physical burden first and third in strength centrality, consistent with their role as the hubs around which the system is organized. Their predictability, however, was modest (R^2^ = 0.37 and 0.34 for the psychological and physical dimensions), and because most of this explained variance was carried by the strong edge between the two dimensions rather than by the external correlates, the variance attributable to the measured psychosocial, lifestyle, and demographic nodes individually was small (Figure 3).

This modest predictability was independently corroborated by the machine-learning analysis, which served as a check on the regression that does not impose the proportional-odds functional form. Each somatic dimension was modeled in its three-category form using three algorithms spanning distinct modeling philosophies: an elastic-net penalized regression as an interpretable linear baseline, and random forest and extreme gradient boosting as tree-based ensembles able to capture nonlinearity and interactions. Models were trained on a stratified 70% partition and evaluated on the held-out 30% test set, with all hyperparameters tuned by 10-fold cross-validation within the training partition under a scheme shared across algorithms. The three algorithms converged on closely comparable and modest discrimination, with the area under the curve falling between 0.64 and 0.66 for both dimensions; no algorithm meaningfully outperformed the others, and the tuned ensembles repeatedly selected conservative configurations, indicating little exploitable nonlinearity beyond the additive structure already captured by the regression. Crucially, both permutation importance and SHAP reproduced the same dissociation: bullying exposure ranked first for both dimensions, followed by sex and age for physical burden, whereas school alienation and parental support rose to the highest ranks for psychological burden, with sex falling to fourth (Supplementary Material, Section S8, Tables S19 and S20, Figure S2).

Three analytic families, survey-weighted ordinal regression, a mixed graphical model, and supervised machine learning, therefore converge on the same conclusion: psychosocial systems align with psychological somatic burden, and biological and demographic factors with physical somatic burden; within the machine-learning analysis, permutation importance and SHAP independently reproduced the same ranking.

## 4. Discussion

In this analytic sample, drawn from the nationally representative 2022 Türkiye Child Survey and consisting of adolescents engaged in formal schooling, somatic symptoms were not a uniform individual complaint but were structured along two dimensions and patterned by the psychosocial risk and support surrounding the adolescent. Three findings carry the argument. Somatic symptoms formed two closely correlated but distinguishable dimensions, physical and psychological, rather than the single dimension commonly assumed. The three relational microsystems of family, school, and peers aligned along a single gradient of psychosocial adversity, and somatic burden rose along it. Most centrally, the two somatic dimensions attached to different kinds of risk and protective factors: psychosocial conditions were linked more closely to psychological somatic burden, whereas biological and demographic characteristics were linked more closely to physical somatic burden, a dissociation that emerged consistently across survey-weighted ordinal regression, a mixed graphical model, and machine learning, with permutation importance and SHAP agreeing within the latter. At the same time, all approaches converged on a modest ceiling of individual-level predictability, indicating that the measured risk and protective factors are associated with somatic burden at the population level without determining it for any individual.

The two-dimensional structure itself deserves comment, because it departs from the prevailing measurement consensus. In item-response analyses across 46 countries, the HBSC Symptom Checklist has generally functioned as a largely unidimensional instrument [20], although earlier cross-national work had recovered separable physical and psychological components [3]. In the present sample, the two-factor solution was decisively superior, and the distinction proved consequential rather than cosmetic: had the eight symptoms been collapsed into a single score, the differential associations reported here, such as parental support relating to one dimension but not the other, would have been invisible. We cannot determine from these data whether the sharper separation reflects the Turkish context, the age range surveyed, or features of the response scales, and measurement-invariance work comparing Türkiye with other HBSC countries would be needed to adjudicate. The practical implication, however, is immediate: where the two dimensions are distinguishable, analyses that preserve them can detect system-specific patterns that a total score conceals.

The clearest such pattern concerned the relational microsystems. Peer victimization was the strongest psychosocial correlate of both dimensions, but its association was larger for psychological than for physical burden, and in the network it connected to psychological burden far more strongly after conditioning on all other variables. This is consistent with meta-analytic evidence that victimization is linked to both somatic and internalizing outcomes [16,17], and it sharpens that evidence by showing where, within the somatic domain, the association concentrates. Parental support showed the same selectivity in a protective direction: it was associated with lower psychological burden but was unrelated to physical burden, echoing reviews that tie the family environment to adolescent adaptation and pain-related outcomes [18,19]. School alienation occupied an intermediate position, associated modestly with both dimensions. Read through an ecological lens [8], the relational microsystems are associated chiefly with the psychological face of somatic burden. These associations are likely to run in both directions. Adolescents who feel persistently unwell may withdraw from peers and from school, becoming both more visible as targets and less able to draw on the support otherwise available to them, while victimization and alienation may in turn intensify symptoms. The cross-sectional design cannot separate these paths, and the smaller but genuine association of peer victimization with physical burden is compatible with either reading.

The complementary pattern anchored the physical dimension. Being female and having a chronic illness were associated with higher burden on both dimensions but more strongly on the physical one, and age increased both in a strictly linear fashion. The female excess in somatic and internalizing symptoms during adolescence is among the most replicated findings in the field [70], and its presence here on both dimensions, yet with a physical preponderance, suggests that sex differences in this national sample are not reducible to the psychological pathway alone. Chronic illness, when treated as a predominantly physical correlate, is biologically expected and serves, in effect, as a positive control: the model recovers a known bodily association where one should exist.

Equally informative is what was absent. Body mass index showed no association with either dimension, and generalized additive models found no U-shaped relationship of the kind reported in recent large-scale work on body mass index and mental health [71]; in this sample the null was flat rather than masked by nonlinearity. Body mass index is in fact the clearest case of convergence between the methods: it showed no independent association with either dimension in the regression, it retained no edge in the network, and it fell outside the eight most important predictors in both machine-learning models. Household income strain and housing problems, although correlated with each other, were not independently associated with either somatic dimension and connected only to each other in the network. One reading, consistent with the broader literature on social determinants [4], is that the association between distal socioeconomic conditions and adolescent somatic health runs largely through the proximal relational microsystems rather than directly, a mediation hypothesis these cross-sectional data can motivate but not test. The lifestyle indicators told a similar story of separation: they were mutually near-orthogonal, formed no latent profiles, and clustered among themselves rather than with the somatic dimensions. The positive associations of organized sport with both dimensions, and of physical activity with psychological burden, run counter to a simple protective account and are not explained by the physical route one might first assume. Conditioning on the psychological dimension removes its association with physical burden entirely, while its association with psychological burden remains (Supplementary Material, Table S18). This points instead to a psychological route, one that might involve competitive or performance pressure, anxiety about being evaluated, disrupted sleep, or heightened bodily awareness. The direction is also open, since adolescents who feel unwell may pull back from organized sport just as readily as they are drawn into it. Reverse causality and residual confounding cannot be ruled out. The present cross-sectional data cannot distinguish among these, and the finding is reported as a question rather than a conclusion.

Before drawing these strands together, one alternative reading deserves to be set out in full, because it is the strongest challenge to the interpretation offered here. The four items that define the psychological dimension, feeling low, irritability, nervousness, and difficulty sleeping, overlap in content with what is ordinarily called internalizing distress, a construct that is itself defined inconsistently across instruments [5]. On this reading, the stronger association of the relational microsystems with the psychological dimension would reflect that overlap rather than any substantive difference between two forms of somatic burden. A second and more general concern points in the same direction. Every construct examined here was reported by the same adolescent at a single point in time, and self-report measures of adversity and of bodily complaint both carry a component of negative affectivity, so that correlations between them are liable to overestimate the true association [72]. Neither possibility can be excluded on cross-sectional, single-informant data, and we do not attempt to exclude them.

What counts against this reading is the shape of the pattern rather than its strength. Discriminant validity is established not by a low correlation between two constructs, which may well be substantial, but by their relating differently to variables external to them [73]. Negative affectivity, on the account that raises the concern, operates as a general nuisance factor rather than a selective one [72]. A general inflation would not, by itself, be expected to produce a specific dissociation. Content overlap and shared negative affectivity would be expected to inflate the paths running from the psychosocial microsystems to the psychological dimension while leaving the remaining paths largely unaffected. They do not readily explain the physical preponderance of female sex, which emerges during puberty alongside changes in pain sensitivity and pain processing rather than in affect [13], nor the predominantly physical attachment of chronic illness, which the preceding paragraphs describe as a positive control. Nor do they readily explain why peer victimization retains an independent association with physical burden. The dissociation is therefore unlikely to be wholly attributable to the content of the psychological items, although content overlap and shared method variance may well contribute to its magnitude.

Showing that the dissociation is real is not the same as showing that its two dimensions can be used as distinct measures. On this second question, the bifactor model is informative, and here our results converge with the largest existing analysis of this scale. As noted above, item-response work across the HBSC countries found the HBSC Symptom Checklist best treated as essentially unidimensional, with departures from a single dimension negligible in most settings [20]. Our bifactor indices tell the same story from a different sample: a strong general factor carries most of the reliable variance in the eight complaints, and once it is removed, the physical and psychological subscale scores retain little of their own. The practical implication is the same. The total score functions as a parsimonious summary of overall symptom burden, whereas the physical and psychological subscales should not be read as independent or pure domain-specific measures; they are strongly overlapping expressions of a dominant general symptom burden. One item bears this out with unusual clarity. Difficulty sleeping, though grouped with the psychological complaints here and in earlier two-factor work on the scale [74], carried no variance specific to that cluster; disturbed sleep behaves instead as a general marker of somatic distress, which is consistent with evidence that sleep problems and internalizing symptoms are reciprocally rather than unidirectionally related in adolescents [75].

None of this dissolves the dissociation we report, because that dissociation was never a property of the subscale scores. Two-factor structure has been recovered repeatedly in this checklist, and the two dimensions have been read as potentially distinct in origin rather than merely as correlated indices [76]. Our contribution is to show where the distinction resides: not in the reliable variance of separate scores, but in how the two dimensions attach to the world outside the checklist, a difference that persisted in the bifactor MIMIC model with the shared factor removed. This locates the kind of validation the measure still needs. Because the dissociation is carried by external relations, and because scalar invariance of this scale does not hold across countries, with the psychological items among the least invariant [20], any cross-national comparison of these dimensions must be anchored in the general factor and in the differential correlates rather than in the subscale scores. Formal invariance testing is the natural next step, and where non-invariance is found, methods that isolate its influence on the structural estimates rather than discarding the affected items offer a way to preserve the comparison [77].

Taken together, the results give empirical content to the framing of somatic symptoms as patterned by psychosocial risk and support. In the network, the two somatic dimensions were among the most central nodes, bound by a strong shared core yet reaching into different regions of the system, which is the topology one would expect if bodily complaints were associated with strain arising in distinct layers of an adolescent’s ecology [6,7]. Yet the same analyses impose discipline on that claim: network predictability and out-of-sample classification converged on modest values, so the measured risk and protective factors pattern somatic burden at the population level without coming close to determining it for any individual. The pattern, in other words, is legible in aggregate and noisy in the particular, which is the configuration in which population-level monitoring and psychosocially targeted intervention are most defensible, and individual-level prediction least so.

These observations suggest practical implications for the disciplines that meet these symptoms first, though each remains provisional given the cross-sectional evidence. For physiotherapists, who increasingly serve as first-contact clinicians for adolescent pain [15], the dissociation supports situating recurrent physical complaints within their psychosocial context, particularly when psychological somatic burden is elevated, rather than confining assessment to the musculoskeletal examination; asking a young person with recurrent headache or backache about their experience of school and peer treatment may, on this evidence, help to locate the complaint within its wider system, an implication that would require longitudinal validation. For schools, the prominence of peer victimization and school alienation is consistent with the rationale for preventive, school-based mental health programs that address the relational climate alongside symptom management [14]. For health systems, the recurring national child survey from which these data derive could, in principle, support population monitoring. Tracked over successive waves, and subject to longitudinal and measurement-invariance validation, the total eight-item checklist might provide a parsimonious population indicator of overall adolescent symptom burden, supporting early identification and the planning of prevention and mental health services, in line with systems approaches to health system strengthening [9].

The study’s strengths include its derivation from a nationally representative survey, the use of sampling weights for population-level estimates, evaluation of the measurement structure before substantive modeling, and convergence across complementary analytic approaches, with the measurement results robust to a full-information sensitivity analysis. Its limitations follow mainly from design. The data are cross-sectional, so no directional or causal claims are warranted. All psychosocial and symptom constructs are self-reported by the same informant, and shared reporting tendencies may inflate associations between psychosocial exposures and psychological symptoms in particular, which counsels caution before reading the dissociation as entirely substantive. The microdata lacked stratum and cluster identifiers, so design-based variance estimation was approximate, although the core associations and path differences proved robust to plausible assumed design effects; peer victimization was strongly skewed, with most adolescents reporting none; and the recoding of unordered response options onto frequency scales, although transparent, is a survey-specific feature. Longitudinal waves of the same survey would allow the mediation and directionality questions raised here to be tested directly, and formal invariance testing would establish whether the two-dimensional somatic structure is a stable feature of the Turkish context.

Two limitations bear directly on the scope of these claims. The first concerns who is represented. Missing data fell almost entirely on the school items and disproportionately on older adolescents, in a pattern consistent with departure from formal education (Section 2.3). The school alienation association is therefore estimated among adolescents still engaged in schooling, and multiple imputation, while it reproduces every estimate, cannot restore a school experience for adolescents to whom the items did not apply. These findings should not be generalized without qualification to all adolescents aged 13 to 17, and least of all to those outside the school system, whose psychosocial configuration may differ in ways this survey cannot show. The second concerns what was measured. The school alienation and parental support scales are shortened, data-derived versions of their source modules. They are internally coherent and behave as expected in response to external variables, but they should not be assumed interchangeable with the complete instruments, and direct comparability testing would be needed before they are treated as such.

## 5. Conclusions

Among the adolescents represented in this analytic sample, somatic symptoms comprise a dominant general burden, alongside two closely related physical and psychological dimensions that show differential external associations. Psychological somatic burden tracks the relational microsystems of peers, school, and family, where peer victimization acts as a risk factor and parental support as a source of resilience, whereas physical somatic burden tracks bodily and demographic characteristics. The practical implications follow, but they require validation before they can be acted on. A clinician seeing an adolescent with recurrent headaches alongside irritability and low mood may have reason to ask about bullying and about feelings of alienation at school. A school facing a rise in psychological and somatic complaints may have reason to look at its relational climate, not only at its health services. And a statistical agency already fielding the child survey may, in eight short items, hold the basis of a recurring population indicator. Whether these eight items can carry that weight is an empirical question this study does not answer. It would take longitudinal evidence that the associations run in the direction assumed, together with measurement-invariance testing, to show that the dimensions mean the same thing across groups and over time. None of these uses requires individual prediction, which these data show to be limited. They ask only that somatic symptoms be read in their psychosocial context rather than in isolation. Whether the associations reported here reflect processes running from the environment to the body, from the body to the environment, or both, is a question these cross-sectional data cannot settle and that longitudinal work should address.

## Figures and Tables

**Table 1:** Weighted characteristics of the analytic sample (*n* = 3523).

Characteristic	Mean (SD)	Median [Min–Max]	% (*n*)
Age, years	14.86 (1.40)	15 [13–17]	
Sex			
Male			52.1 (1864)
Female			47.9 (1659)
Somatic symptom category			
Low			40.6 (1402)
Moderate			29.8 (1076)
High			29.6 (1045)
**Lifestyle and health**			
Body mass index, z-score	0.00 (0.99)	−0.17 [−2.59, 5.73]	
Healthy diet score	4.97 (1.04)	5.00 [0, 6]	
Unhealthy diet score	4.39 (1.25)	4.67 [0, 6]	
Physical activity, days/week	1.24 (2.08)	0 [0, 7]	
Organized sport participation (yes)			21.4 (776)
Chronic illness (yes)			10.8 (377)
**Socioeconomic context**			
Household income strain	3.72 (0.92)	4 [1, 5]	
Housing problems score	0.20 (0.24)	0.20 [0, 1]	

Survey-weighted to the population represented by the analytic sample, which consists predominantly of school-attending adolescents. Continuous variables, weighted mean (SD) and median [minimum, maximum]; categorical variables, weighted percentage (unweighted *n*). Higher diet and housing scores indicate more frequent intake and poorer conditions, respectively; income strain ranges from 1 (very easily) to 5 (with great difficulty). SD, standard deviation.

**Table 2:** Confirmatory factor analysis of the measurement constructs: standardized loadings, standard errors, test statistics, internal consistency, and model fit.

	Item	Std. *β*	SE	*z*	*p*	Reliability
**Somatic symptoms**	**Physical somatic factor**					
	Headache	0.757	0.013	60.47	<0.001	*α* = 0.791 *ω* = 0.793
	Stomachache	0.668	0.014	46.31	<0.001	
	Backache	0.706	0.015	47.51	<0.001	
	Dizziness	0.695	0.019	37.21	<0.001	
	**Psychological somatic factor**					
	Feeling low	0.805	0.008	104.52	<0.001	α = 0.856 ω = 0.867
	Irritability	0.893	0.006	142.54	<0.001	
	Feeling nervous	0.890	0.006	141.62	<0.001	
	Difficulties falling asleep	0.576	0.017	33.83	<0.001	
	*Factor correlation (r = 0.733) [95% CI 0.707, 0.758]*					
**School alienation**	Feeling excluded or left out	0.699	0.010	70.56	<0.001	*α* = 0.805 *ω* = 0.808
	Feeling awkward and out of place	0.750	0.009	81.94	<0.001	
	Feeling lonely at school	0.838	0.008	102.79	<0.001	
**Parental support**	(a) Parents help me as much as I need	0.799	0.007	107.50	<0.001	*α* = 0.918 *ω* = 0.919
	(b) Parents let me do things I like	0.739	0.008	93.49	<0.001	
	(c) Parents show they care about me	0.872	0.005	166.41	<0.001	
	(d) Parents try to understand my worries	0.883	0.005	175.70	<0.001	
	(e) Parents encourage me to decide	0.801	0.007	118.28	<0.001	
	(f) Parents make me feel better when upset	0.727	0.009	83.69	<0.001	
	*Residual covariances 1: Help (a) ↔ Care (c) (0.252)*					
	*Residual covariances 2: Understand (d) ↔ Encourage (e) (0.190)*					
**Peer victimization**	Deliberately left out	0.828	0.014	57.98	<0.001	*α* = 0.930 *ω* = 0.930
	Made fun of	0.878	0.012	73.48	<0.001	
	Threatened	0.872	0.022	40.13	<0.001	
	Belongings taken or destroyed	0.804	0.022	36.78	<0.001	
	Pushed around	0.871	0.016	54.25	<0.001	
	Rumors spread	0.752	0.017	43.01	<0.001	
	**Model comparison and fit**	**CFI**	**TLI**	**RMSEA [90% CI]**		**SRMR**
	Somatic, one-factor	0.954	0.936	0.132 [0.126, 0.139]		0.077
	Somatic, two-factor	0.987	0.981	0.073 [0.066, 0.079]		0.046
	Somatic, bifactor	0.998	0.995	0.037 [0.029, 0.046]		0.019
	Parental support	0.994	0.987	0.098 [0.088, 0.109]		0.018
	Peer victimization	0.985	0.975	0.054 [0.044, 0.063]		0.035
	School alienation, single-factor	just-identified (*df* = 0)				

Std. β, standardized factor loading; SE, standard error; *z*, Wald *z* statistic; α, ordinal coefficient alpha; ω, McDonald’s omega; CFI, comparative fit index; TLI, Tucker–Lewis index; RMSEA, root mean square error of approximation; SRMR, standardized root mean square residual; *df*, degrees of freedom; CI, confidence interval; r, correlation between latent factors. Letters (a)–(f) label the six parental support items and identify the item pairs referenced in the residual covariance rows. All models were estimated with the WLSMV estimator treating items as ordinal, with school alienation reverse-scored so that higher values indicate greater alienation. Fit statistics for the one-factor, two-factor, and bifactor somatic models are reported above; the two-factor model was retained over the one-factor model, and the bifactor model, which fitted best as a more flexible model generally does, was interpreted through its loadings and bifactor indices rather than its fit (Supplementary Material, Section S2, Table S7). The school alienation factor is just-identified, so global fit indices are not estimable. Item-selection details and the full validity analysis are reported in the Supplementary Material, Section S2.

**Table 3:** Latent-variable model of the two somatic dimensions and formal tests of differential association (*n* = 3523).

	Physical Somatic Burden		Psychological Somatic Burden		Test of Differential Association		
Predictor	β	*p*	β	*p*	Δβ (SE)	z	*p*
**Psychosocial microsystems**							
Peer victimization	0.210	<0.001	0.273	<0.001	−0.062 (0.021)	−2.97	0.003
School alienation	0.081	0.001	0.129	<0.001	−0.047 (0.025)	−1.91	0.057
Parental support	−0.026	0.268	−0.103	<0.001	0.076 (0.022)	3.53	<0.001
**Lifestyle and health**							
BMI z-score	−0.020	0.385	−0.002	0.904	−0.018 (0.023)	−0.77	0.441
Healthy diet	−0.121	<0.001	−0.079	<0.001	−0.042 (0.022)	−1.91	0.056
Unhealthy diet	0.006	0.775	0.013	0.511	−0.007 (0.021)	−0.32	0.747
Physical activity	0.028	0.244	0.057	0.008	−0.028 (0.024)	−1.17	0.242
Chronic illness	0.364	<0.001	0.171	0.010	0.193 (0.065)	2.97	0.003
Organized sport	0.190	0.001	0.273	<0.001	−0.083 (0.058)	−1.44	0.150
**Socioeconomic context**							
Income strain	0.019	0.420	0.034	0.110	−0.016 (0.022)	−0.70	0.483
Housing problems	0.042	0.086	−0.005	0.815	0.047 (0.023)	2.04	0.042
**Sociodemographic**							
Age	0.151	<0.001	0.149	<0.001	0.002 (0.022)	0.08	0.938
Sex (female)	0.641	<0.001	0.422	<0.001	0.218 (0.047)	4.64	<0.001
**Omnibus Wald tests of the equality of paths across the two dimensions**					**χ^2^**	**df**	***p***
All 13 paths					83.74	13	<0.001
Psychosocial microsystems					31.86	3	<0.001
Biological–demographic					31.34	2	<0.001
Four core paths ^a^					54.14	4	<0.001
**Model summary**	***χ^2^*** **(97)**	**CFI**	**TLI**	**RMSEA**	**SRMR**	**Residual *r*** ^b^	**R^2^** ^c^
Estimate	522.83	0.977	0.993	0.035	0.044	0.686	0.179/0.179

β, unstandardized coefficient; Δβ, difference between the physical and psychological coefficients (physical minus psychological); SE, standard error; *z*, Wald *z* statistic; ***χ^2^***, chi-square test statistic; *df*, degrees of freedom; BMI, body mass index; CFI, comparative fit index; TLI, Tucker–Lewis index; RMSEA, root mean square error of approximation; SRMR, standardized root mean square residual. Coefficients are from a single survey-weighted multiple-indicator multiple-cause model; each difference was tested by a Wald test of the corresponding equality constraint. All estimates are cross-sectional associations and do not support causal or temporal interpretation. Sensitivity analyses under alternative estimation, categorization, imputation, and assumed design-effect schemes are reported in Supplementary Material, Sections S1, S5 and S6. ^a^ Peer victimization, parental support, sex, and chronic illness tested jointly. ^b^ Correlation between the latent residuals of the two dimensions; SE = 0.016, *p* < 0.001. ^c^ Physical and psychological dimensions, respectively.

## Data Availability

The microdata analyzed in this study were obtained from the Turkish Statistical Institute (TURKSTAT) and are not publicly available owing to confidentiality restrictions; researchers may apply through TURKSTAT’s official data access procedures. The analysis code and aggregate outputs are openly available on GitHub (R 4.5.2.) (https://github.com/sy142/adolescent-somatic-systems, accessed on 22 July 2026) and archived on Zenodo (https://doi.org/10.5281/zenodo.20631358).

## Supplementary Materials

The following supporting information can be downloaded at: https://www.mdpi.com/article/10.3390/children13080985/s1, Section S1: Missing Data, Multiple Imputation, and the Analytic Sample; Table S1: Missing data patterns and characteristics of included versus excluded participants; Table S2: Odds ratios pooled across 30 imputed datasets compared with the complete-case estimates. Section S2: Confirmatory Measurement Models, Item Selection, and Replication; Table S3: Confirmatory factor model comparison and item selection for the multi-item constructs; Table S4: Convergent and discriminant validity of the measurement factors; Table S5: Five-factor measurement model containing all multi-item constructs; Table S6: Replication of the measurement decisions in two random halves of the analytic sample; Table S7: Bifactor model of the eight HBSC-SCL somatic items: model comparison, standardized loadings with bifactor indices, and bifactor MIMIC regressions. Section S3: Multiple Correspondence Analysis of the Psychosocial Microsystems; Table S8: Category distributions of the three psychosocial domains entered into the multiple correspondence analysis; Table S9: Category coordinates, contributions, and squared correlations from the multiple correspondence analysis; Table S10: Ordinal trend between each psychosocial domain and somatic symptom category. Section S4: Latent Profile Analysis of Lifestyle and Socioeconomic Indicators; Figure S1: Pearson correlations among the six lifestyle and socioeconomic indicators; Table S11: Fit indices for latent profile models fitted to the lifestyle and socioeconomic indicators. Section S5: Ordinal Regression and Robustness of the Outcome Specification; Table S12: Ordinal logistic regression coefficients for physical and psychological somatic burden; Table S13: Associations with each somatic dimension under the primary and five alternative specifications of the outcome and of the psychosocial predictors; Table S14: *p*-values for each association under progressively inflated standard errors. Section S6: Latent-Variable Model and Formal Tests of Differential Association; Table S15: Standardized coefficients and the physical-minus-psychological difference under three estimation schemes; Table S16: Sensitivity of the latent path-difference tests to the unobserved sampling design: *p*-values under progressively inflated standard errors. Section S7: Network Structure of Somatic Dimensions and Their Correlates; Table S17: Bootstrap-validated network edges involving the somatic dimensions, grouped by neighbor system; TableS18: Conditional models reconciling the regression and the network. Section S8: Machine-Learning Validation of Predictor Importance; Table S19: Cross-validated and held-out classification performance of the three algorithms; Table S20: Predictor importance for the high somatic category (mean absolute SHAP); Figure S2: SHAP summary for the gradient-boosting model. Section S9: Statistical Software and Reproducibility; Table S21: R packages used for statistical analysis. References [78,79,80,81,82,83] are cited in the Supplementary Materials.

## Abbreviations

The following abbreviations are used in this manuscript:

BMI	Body mass index
CFI	Comparative fit index
CI	Confidence interval
HBSC	Health Behavior in School-aged Children
HBSC-SCL	HBSC Symptom Checklist
LASSO	Least absolute shrinkage and selection operator
MCA	Multiple correspondence analysis
MGM	Mixed graphical model
RMSEA	Root mean square error of approximation
SD	Standard deviation
SE	Standard error
SHAP	Shapley additive explanations
SRMR	Standardized root mean square residual
TCA	Türkiye Child Survey (Türkiye Çocuk Araştırması)
TLI	Tucker–Lewis index
TUBITAK	Scientific and Technological Research Council of Türkiye
TURKSTAT	Turkish Statistical Institute
UNICEF	United Nations Children’s Fund
WHO	World Health Organization
WLSMV	Weighted least squares mean and variance adjusted estimator
