# Supplementary material for: Adolescent Somatic Symptoms Under Psychosocial Adversity: Differential Links of Risk and Protective Factors to Physical and Psychological Burden in Türkiye
Source: Children (Basel). 2026 Jul 24;13(8):985. doi: 10.3390/children13080985 (PMC13510805; doi:10.3390/children13080985)
Supplement: Supplementary file 1 [file children-13-00985-s001.zip › children-4417959-supplementary.pdf]

Supplementary Information for:

Adolescent Somatic Symptoms Under Psychosocial Adversity: Differential Links of Risk and Protective Factors to Physical and Psychological Burden in Türkiye

Derya Azim, Sevde Betül Kara, Muhammed Emre Güvey, Ecenur Aydemir, Sümeyra Gündem, Salim Yılmaz

Corresponding author: Salim Yılmaz,
Email: salim.yilmaz@acibadem.edu.tr

Supplementary Materials

Adolescent Somatic Symptoms Under Psychosocial Adversity: Differential Links of Risk and Protective Factors to Physical and Psychological Burden in Türkiye

This supplementary material provides extended methodological detail and supporting analyses for the study examining how adolescent somatic symptoms are patterned by psychosocial risk and support in Türkiye. The analysis used microdata from the 2022 Türkiye Child Survey (Turkish Statistical Institute, 2023), a nationally representative cross-sectional survey, drawing on the self-report module administered to adolescents aged 13–17 years. It documents the missing data assessment and analytic sample selection, the confirmatory measurement models, the multiple correspondence and latent profile analyses, the full ordinal regression results, and the network and machine-learning analyses that complement the main findings.

Abbreviations

AIC:	Akaike Information Criterion
AUC:	Area Under the Curve
AVE:	Average Variance Extracted
BIC:	Bayesian Information Criterion
BLRT:	Bootstrap Likelihood Ratio Test
BMI:	Body Mass Index
CFI:	Comparative Fit Index
CI:	Confidence Interval
CR:	Composite Reliability
FIML:	Full-Information Maximum Likelihood
HBSC:	Health Behaviour in School-aged Children
HTMT:	Heterotrait–Monotrait Ratio
ICC:	Intraclass Correlation Coefficient
LPA:	Latent Profile Analysis
MAR:	Missing at Random
MCA:	Multiple Correspondence Analysis
MCAR:	Missing Completely at Random
MGM:	Mixed Graphical Model
OR:	Odds Ratio
RMSEA:	Root Mean Square Error of Approximation
saBIC:	Sample Size-Adjusted Bayesian Information Criterion
SD:	Standard Deviation
SHAP:	SHapley Additive exPlanations
SRMR:	Standardized Root Mean Square Residual
TLI:	Tucker–Lewis Index
TURKSTAT:	Turkish Statistical Institute
WLSMV:	Weighted Least Squares Mean- and Variance-Adjusted

Contents and List of Supplementary Tables and Figures

Section S1: Missing Data, Multiple Imputation, and the Analytic Sample 1

Table S1. Missing data patterns and characteristics of included versus excluded participants........ 1

Table S2. Odds ratios pooled across 30 imputed datasets compared with the complete-case estimates........ 2

Section S2: Confirmatory Measurement Models, Item Selection, and Replication 4

Table S3. Confirmatory factor model comparison and item selection for the multi-item constructs 5

Table S4. Convergent and discriminant validity of the measurement factors........ 5

Table S5. Five-factor measurement model containing all multi-item constructs........ 6

Table S6. Replication of the measurement decisions in two random halves of the analytic sample 7

Table S7. Bifactor model of the eight HBSC-SCL somatic items: model comparison, standardized loadings with bifactor indices, and bifactor MIMIC regressions........ 9

Section S3: Multiple Correspondence Analysis of the Psychosocial Microsystems........11

Table S8. Category distributions of the three psychosocial domains entered into the multiple correspondence analysis........11

Table S9. Category coordinates, contributions, and squared correlations from the multiple correspondence analysis........11

Table S10. Ordinal trend between each psychosocial domain and somatic symptom category 12

Section S4: Latent Profile Analysis of Lifestyle and Socioeconomic Indicators 13

Figure S1. Pearson correlations among the six lifestyle and socioeconomic indicators........ 13

Table S11. Fit indices for latent profile models fitted to the lifestyle and socioeconomic indicators........ 14

Section S5: Ordinal Regression and Robustness of the Outcome Specification 15

Table S12. Ordinal logistic regression coefficients for physical and psychological somatic burden 15

Table S13. Associations with each somatic dimension under the primary and five alternative specifications of the outcome and of the psychosocial predictors........ 16

Table S14. p-values for each association under progressively inflated standard errors........ 17

Section S6: Latent-Variable Model and Formal Tests of Differential Association........ 19

Table S15. Standardized coefficients and the physical-minus-psychological difference under three estimation schemes........ 19

Table S16. Sensitivity of the latent path-difference tests to the unobserved sampling design: p-values under progressively inflated standard errors........ 21

Section S7: Network Structure of Somatic Dimensions and Their Correlates 22

Table S17. Bootstrap-validated network edges involving the somatic dimensions, grouped by neighbor system........ 23

Table S18. Conditional models reconciling the regression and the network 24

Section S8: Machine-Learning Validation of Predictor Importance 26

Table S19. Cross-validated and held-out classification performance of the three algorithms 26

Table S20. Predictor importance for the high somatic category (mean absolute SHAP) 27

Figure S2. SHAP summary for the gradient-boosting model........ 28

Section S9: Statistical Software and Reproducibility........ 29

Table S21. R packages used for statistical analysis........ 29

References for Supplementary Material 30

Section S1: Missing Data, Multiple Imputation, and the Analytic Sample

The derived dataset comprised 4072 adolescents aged 13–17 years who completed the self-report module of the 2022 Türkiye Child Survey (Turkish Statistical Institute, 2023). The subsequent analytic chain, which comprises confirmatory factor analysis, multiple correspondence analysis, latent profile analysis, and ordinal regression with machine-learning comparison, relies on a common set of indicators. We therefore first characterized the extent and structure of missingness across all analytic variables and assessed whether restricting the analysis to participants with complete data would introduce selection bias. Table S1 presents the variable-level missing data, the distribution of complete versus incomplete cases, and a comparison of the demographic, socioeconomic, and outcome characteristics of included versus excluded participants.

Table S1. Missing data patterns and characteristics of included versus excluded participants

Panel A. Missing values by variable (N = 4072)	**n missing**	**% missing**	
School alienation: feeling excluded	538	13.21	
School alienation: feeling lonely	536	13.16	
School belonging index	473	11.62	
School alienation: feeling awkward	464	11.39	
BMI z-score	7	0.17	
Chronic illness	1	0.02	
All somatic symptom items and scores	0	0.00	
Peer victimization	0	0.00	
All parental support items	0	0.00	
Healthy diet, unhealthy diet, physical activity, organized sport	0	0.00	
Household income strain, housing problems	0	0.00	
Sex, age	0	0.00	
Panel B. Included versus excluded participants	**Included (n = 3523)**	**Excluded (n = 549)**	***p***
**Age, years, mean (SD)**	14.9 (1.4)	15.7 (1.2)	**<0.001**
**Age-group, n (%)**			
13 years	753 (21.4)	31 (5.6)	
14 years	742 (21.1)	74 (13.5)	
15 years	769 (21.8)	112 (20.4)	
16 years	686 (19.5)	144 (26.2)	
17 years	573 (16.3)	188 (34.2)	
**Aged 16 or 17, combined**	**1259 (35.7)**	**332 (60.5)**	
**Sex, n (%)**			**0.007**
Male	1864 (52.9)	256 (46.6)	
Female	1659 (47.1)	293 (53.4)	
**Somatic category, n (%)**			0.196
Low	1402 (39.8)	197 (35.9)	
Moderate	1076 (30.5)	174 (31.7)	
High	1045 (29.7)	178 (32.4)	
**Total somatic burden, mean (SD)**	0.8 (0.7)	0.9 (0.8)	0.144
**Household income strain, mean (SD)**	3.7 (0.9)	3.9 (0.9)	**<0.001**
**BMI z-score, mean (SD)**	0.0 (1.0)	0.0 (1.1)	0.527
**Peer victimization, mean (SD)**	0.2 (0.4)	0.2 (0.5)	0.867
**Parental relationship, mean (SD)**	3.8 (0.5)	3.7 (0.5)	**<0.001**

Data are mean (SD) or n (%). p-values are derived from independent-samples t tests for continuous variables and χ^2 tests for categorical variables. Continuous comparisons were confirmed with Mann–Whitney U tests. Age-group percentages are column percentages and are shown for description; the difference in age is tested in the row above. Excluded adolescents were markedly older, with 60.5% aged 16 or 17 compared with 35.7% of those included, consistent with the concentration of missing values in the school items and with the departure of older adolescents from formal schooling.

Missing data were concentrated in the items underlying the school constructs. The three school alienation items and the school belonging index each had missing rates between 11 and 13 percent (feeling excluded, 13.21%; feeling lonely, 13.16%; school belonging index, 11.62%; feeling awkward, 11.39%), reflecting the shared scoring rule under which "not applicable" responses were treated as missing. The remaining variables were either complete or near-complete, with only the BMI z-score (0.17%) and chronic illness (0.02%) showing any missingness. In total, 3523 participants (86.52%) had complete data on all analytic variables, and 549 (13.48%) had at least one missing value.

Little's test indicated that the data were not missing completely at random ($\chi^2 = 468.23$, df = 180, $p < 0.001$) (Little, 1988), distributed across 11 missing data patterns. To characterize the nature of this missingness, we compared participants included in versus excluded from the complete-case analytic sample. Excluded participants were significantly older (15.7 vs 14.9 years, $p < 0.001$), more likely to be female (53.4% vs 47.1%, $p = 0.007$), reported greater household income strain (3.9 vs 3.7, $p < 0.001$), and reported slightly less positive parental relationships (3.7 vs 3.8, $p < 0.001$). Critically, the two groups did not differ on the primary outcome: the distribution of somatic category ($p = 0.196$) and the mean total somatic burden ($p = 0.144$) were equivalent across inclusion status, as were the BMI z-score ($p = 0.527$) and bullying exposure ($p = 0.867$). These patterns indicate that missingness was related to observed demographic and socioeconomic characteristics rather than to the outcome itself. This pattern most plausibly arises because older adolescents and those outside conventional schooling were more likely to answer school-related items as "not applicable." The missingness is therefore consistent with a missing-at-random mechanism with respect to somatic burden, although this assumption cannot be verified from the observed data.

Because the analytic chain integrates a geometric multivariate method (multiple correspondence analysis), a person-centered mixture model (latent profile analysis) and machine-learning models that propagate derived scores across stages and do not accommodate full-information estimation, we adopted a single complete-case analytic sample (n = 3523) for all analyses to ensure a consistent sample size and identical case base across the analytic chain. The absence of any difference in somatic burden between included and excluded participants suggests that complete-case analysis introduces minimal bias with respect to the study outcome, although the findings are best generalized to adolescents engaged in formal schooling. As a sensitivity check, the measurement models were re-estimated on the full sample under full-information maximum likelihood, yielding substantively identical factor structures and loadings (Section S2).

As a further check on the consequences of complete-case analysis, we repeated the primary regression models on the full survey file using multiple imputation. Missing values were imputed at item level by predictive mean matching (30 imputations, five iterations), with all analytic variables and the sampling weight included in the imputation model. Subscale scores, outcome categories and standardized predictors were reconstructed within each imputed dataset, the two survey-weighted ordinal regressions were refitted, and the estimates were combined by Rubin's rules (Rubin, 1987). The pooled estimates for the full sample of 4072 adolescents are compared with the complete-case estimates in Table S2.

Table S2. Odds ratios pooled across 30 imputed datasets compared with the complete-case estimates

Predictor	Physical somatic burden		Psychological somatic burden	
	Complete case (n = 3523)	Imputed (N = 4072)	Complete case (n = 3523)	Imputed (N = 4072)
**Peer victimization**	**1.429**	**1.437 (<0.001)**	**1.840**	**1.919 (<0.001)**
School alienation	1.121	1.120 (0.002)	1.177	1.186 (<0.001)
**Parental support**	**1.001**	**1.003 (0.921)**	**0.883**	**0.899 (0.003)**
BMI z-score	0.958	0.985 (0.641)	0.988	0.971 (0.363)
Healthy diet	0.842	0.856 (<0.001)	0.860	0.879 (<0.001)
Unhealthy diet	1.004	0.996 (0.899)	1.041	1.051 (0.125)
Physical activity	1.061	1.059 (0.080)	1.114	1.094 (0.005)
**Chronic illness**	**1.612**	**1.604 (<0.001)**	**1.374**	**1.388 (0.004)**
**Organized sport**	**1.421**	**1.412 (<0.001)**	**1.573**	**1.542 (<0.001)**
Income strain	0.988	1.012 (0.719)	1.044	1.038 (0.260)
Housing problems	1.054	1.046 (0.190)	0.968	0.983 (0.627)
Age	1.259	1.276 (<0.001)	1.205	1.221 (<0.001)
**Sex (female)**	**2.351**	**2.266 (<0.001)**	**1.820**	**1.785 (<0.001)**

Odds ratios are from survey-weighted ordinal logistic regressions of the three-level somatic categories, adjusted for all 13 predictors. p-values in parentheses are for the imputed estimates. Continuous predictors are standardized; sex, chronic illness and organized sport are binary. Rows in bold are the associations that carry the substantive argument.

No association changed direction, and none crossed the conventional threshold of significance. The differential pattern that the study reports was likewise unchanged: peer victimization and parental support remained more strongly associated with psychological burden, and female sex and chronic illness with physical burden.

Multiple imputation does not, however, resolve the substantive limitation created by this missingness. The missing values are almost entirely confined to the three school alienation items, and those items were not applicable to

adolescents outside formal schooling. Imputing them therefore attributes a school-based experience to adolescents who may not be attending school, and does so on the assumption that the data are missing at random given the observed variables, an assumption that cannot be verified. The imputation is best read as a statistical sensitivity check rather than as a remedy, and the findings remain best generalized to adolescents engaged in formal schooling.

Survey weights and analytic strategy

TURKSTAT provided final person-level sampling weights for the 13–17-year module (FAKTOR_FERT_1317), expanding the analytic sample to a weighted population of approximately 5.57 million adolescents aged 13–17 years; the full survey sample corresponds to 6.46 million, so that the complete-case sample represents 86% of the weighted national population. The weights account for differential selection probabilities, nonresponse, and poststratification to population totals; stratum and primary sampling unit identifiers were not released with the microdata. Because the design could therefore be specified with weights alone, any clustering in the sample is unaccounted for and the standard errors reported throughout are, to that extent, likely to be underestimated (Kish, 1965). The robustness of each association to a range of plausible design effects is examined in Section S5 (Table S14), and that of the latent path-difference tests in Section S6 (Table S16). We applied a hybrid analytic strategy. Measurement, geometric and person-centered models (confirmatory factor analysis, multiple correspondence analysis, and latent profile analysis, respectively) were estimated on unweighted data, because these models recover measurement and configural structure rather than population quantities, and weighting affects the generalization of point estimates rather than the recovered structure. All population-level quantities, including descriptive prevalences and the final associations relating socioeconomic-lifestyle configurations and psychosocial position to somatic burden, were estimated with the sampling weight applied through a survey design object, so that these estimates generalize to the population represented by the analytic sample, which consists predominantly of adolescents attending school.

In the unweighted analytic sample (n = 3523), the mean age was 14.88 years (SD 1.38; median 15 [13, 17]), and 52.9% (n = 1864) were male and 47.1% (n = 1659) female. Somatic symptom category was distributed as 39.8% low (n = 1402), 30.5% moderate (n = 1076), and 29.7% high (n = 1045). The mean body mass index z-score was 0.00 (SD 0.99; median −0.16 [−2.59, 5.73]). Adolescents reported a mean healthy diet score of 4.96 (SD 1.04), a mean unhealthy diet score of 4.38 (SD 1.26), and a mean of 1.26 days (SD 2.09) of at least 60 minutes of physical activity in the previous week; 22.0% (n = 776) took part in organized sport and 10.7% (n = 377) reported a chronic illness. Mean household income strain was 3.72 (SD 0.93) and the mean housing problems score was 0.20 (SD 0.24). These unweighted figures correspond closely to the weighted estimates reported in the main text (Table 1).

Section S2: Confirmatory Measurement Models, Item Selection, and Replication

The multi-item self-report constructs in this study, somatic symptom burden, school alienation, perceived parental support, and peer victimization, were measured with items already included in the 2022 Türkiye Child Survey, which adapted them from established instruments including the Health Behavior in School-aged Children (HBSC) survey, the Psychological Sense of School Membership scale, and the Multidimensional Scale of Perceived Social Support. Rather than developing new instruments, we evaluated the factor structure of these existing items in the present sample and, where a set of items did not form a coherent factor, retained the subset with acceptable measurement properties. All confirmatory factor models were estimated with the WLSMV estimator, which treats items as ordinal and is appropriate for the five-category, positively skewed response distributions observed here. Model fit was judged against the comparative fit index (CFI) and Tucker-Lewis index (TLI), with values of 0.90 or above indicating acceptable fit, and the root mean square error of approximation (RMSEA) and standardized root mean square residual (SRMR), with values of 0.08 or below indicating acceptable fit. We note that the RMSEA is known to be upwardly biased in models with few degrees of freedom combined with large samples, so for the more parsimonious models we interpret it alongside the other indices rather than in isolation (Kenny et al., 2015).

Whereas the eight somatic symptom items were retained in full (their two-factor structure is reported in the main text, Table 2), two of the other multi-item constructs required item selection before a coherent factor could be retained; these paths are summarized in Table S3. For the school experience, the survey included seven items, three worded positively (making friends easily, feeling that one belongs, feeling liked by other students) and four worded negatively (feeling excluded, feeling awkward and out of place, feeling lonely, and feeling anxious before examinations). A one-factor model treating all seven items as a single school belonging construct fit the data poorly. Inspection of the item parameters showed that the examination anxiety item was essentially unrelated to the remaining items, consistent with its content, which concerns test anxiety rather than the school social environment, and a model that removed it improved substantially. The item on feeling liked by other students loaded weakly on the positive factor (standardized loading 0.22) and contributed little to internal consistency. Successive models therefore removed the examination anxiety item and then the feeling-liked item. The three negatively worded items concerning exclusion, awkwardness, and loneliness formed a strong, internally consistent factor that we retained as a measure of school alienation. We carried this three-item factor forward rather than a positive school belonging score because it was psychometrically stronger and because the experience of exclusion and loneliness maps more directly onto the socioecological framework, in which adverse experiences within the school microsystem are hypothesized to relate to somatic burden.

The parental support items required a similar reduction. The survey included eight items on the relationship with parents or guardians, six describing supportive behaviors (providing help, allowing valued activities, showing care, understanding worries, encouraging independent decisions, and providing comfort) and two describing parental control (treating the adolescent like a baby and trying to control everything they do). In a one-factor model across all eight items, the two control items failed to load on the support dimension: their standardized loadings were near zero or negative (treated like a baby, −0.10; controlling, 0.03, not significant), and they were essentially uncorrelated with the supportive items in the polychoric matrix. This pattern indicates that parental control and parental support are distinct dimensions rather than two poles of a single construct, consistent with the broader literature distinguishing warmth and support from psychological control. We therefore retained the six supportive items as a measure of perceived parental support. Inspection of modification indices for this six-item model identified two residual covariances with clear content overlap, between the items on receiving help and feeling cared for, and between the items on having one's worries understood and being encouraged to make one's own decisions; these were freed because the paired items share specific wording beyond the general support factor. The resulting model fit well, with all six loadings between 0.73 and 0.88 and high internal consistency ($\alpha = 0.918$, $\omega = 0.919$). The six peer victimization items formed a strong single factor without modification (loadings 0.75 to 0.88), and were retained in full.

Table S3. Confirmatory factor model comparison and item selection for the multi-item constructs

Model	Items	CFI	TLI	RMSEA [90% CI]	SRMR	Decision
Somatic Symptoms (HBSC-SCL)						
One-factor	8	0.954	0.936	0.132 [0.126, 0.139]	0.077	Poor fit
**Two-factor (physical, psychological)**	8	0.987	0.981	0.073 [0.066, 0.079]	0.046	Retained
School Experience Items (school alienation)						
One-factor	7	0.903	0.855	0.154 [0.147, 0.161]	0.075	Poor fit
Two-factor	7	0.960	0.935	0.103 [0.095, 0.111]	0.049	RMSEA above threshold
Two-factor, anxiety item removed	6	0.979	0.960	0.093 [0.084, 0.103]	0.035	Improved
Two-factor, anxiety and liked items removed	5	0.980	0.950	0.124 [0.111, 0.138]	0.031	Positive factor weak
**One-factor school alienation**	3	—	—	Just-identified	—	Retained
Parental Relationship Items (parental support)						
One-factor, all items	8	0.982	0.974	0.109 [0.102, 0.115]	0.043	Two control items do not load; removed
One-factor support, no residual cov.	6	0.986	0.977	0.131 [0.122, 0.141]	0.025	RMSEA inflated by low df
**One-factor support, two residual cov.**	6	0.994	0.987	0.098 [0.088, 0.109]	0.018	Retained
**Peer Victimization (HBSC bullying items)**						
**One-factor**	6	0.985	0.975	0.054 [0.044, 0.063]	0.035	Retained, no modification

CFI, comparative fit index; TLI, Tucker-Lewis index; RMSEA, root mean square error of approximation; SRMR, standardized root mean square residual; CI, confidence interval; df, degrees of freedom.

All models were estimated with the WLSMV estimator treating items as ordinal. Successive rows within each block show the item-selection path described in the text. Just-identified three-indicator factors have no estimable global fit; the school alienation factor is evaluated within the five-factor model reported in Table S5.

We examined the convergent and discriminant validity of the retained factors using composite reliability (CR), average variance extracted (AVE), and, for the two correlated somatic factors, the heterotrait-monotrait ratio of correlations (HTMT) and the Fornell-Larcker criterion; the results are reported in Table S4.

Table S4. Convergent and discriminant validity of the measurement factors

Factor	Items	CR	AVE	√AVE	Interfactor r	HTMT
Physical somatic	4	0.800	0.500	0.707	0.733	0.788
Psychological somatic	4	0.875	0.642	0.802		
School alienation	3	0.808	0.585	0.765	—	—
Parental support	6	0.917	0.649	0.806	—	—
Peer victimization	6	0.932	0.698	0.835	—	—

CR, composite reliability (threshold ≥ 0.70); AVE, average variance extracted (threshold ≥ 0.50); √AVE, square root of AVE, compared against the interfactor correlation for the Fornell-Larcker criterion; HTMT, heterotrait-monotrait ratio of correlations (threshold ≤ 0.85). The interfactor correlation and HTMT apply to the two somatic factors, which were modeled together; the remaining factors were modeled separately and have no discriminant comparison in this table. CR and AVE were computed from the standardized WLSMV loadings.

Composite reliability was high for all five factors (0.80 to 0.93), supporting convergent validity. Average variance extracted exceeded the 0.50 threshold for the psychological somatic factor (0.64), school alienation (0.58), parental support (0.65), and peer victimization (0.70), and reached it for the physical somatic factor (0.50); because the composite reliability of the physical factor was well above 0.60, its convergent validity is considered adequate notwithstanding the borderline AVE (Fornell & Larcker, 1981). For the two correlated somatic factors, the HTMT was 0.79, below the 0.85 threshold, supporting discriminant validity. The Fornell-Larcker criterion was met for the psychological factor, whose square root of AVE (0.80) exceeded the interfactor correlation (0.73), but it was not met for the physical factor (0.71 versus 0.73). The two dimensions plainly share a substantial common core, and their separability does not rest on a low correlation between them, which would be the wrong criterion here. It rests on three other grounds. First, the advantage of the two-factor model over the one-factor model replicates in two independent random halves of the sample, with scaled chi-square differences of 196.01 and 233.53 (Table S6). Second, the correlation between the factors is stable across those halves (0.728 and 0.739). Third, and most directly, the two factors have significantly different external correlates: in a latent model in which both dimensions are regressed simultaneously on all thirteen predictors, the constraint that the paths are equal across dimensions is decisively rejected ($\chi^2(13) = 83.74$, $p < 0.001$; main text Table 3 and Table S15). Following Henseler et al. (2015), who show the Fornell-Larcker criterion to be insensitive relative to the heterotrait-monotrait ratio, and given that

discriminant validity is properly established by differential relations with external variables rather than by the size of the interfactor correlation alone, we describe the two somatic dimensions as closely related but distinguishable.

The measurement models above were estimated construct by construct, in the order in which the constructs enter the analysis. One consequence of that choice requires explicit treatment. The school alienation factor retains three items, and a three-item factor specified alone is just-identified: it has no degrees of freedom, and its global fit therefore cannot be evaluated. To give that factor an evaluable fit, and to inspect the discriminant structure of all constructs at once, we re-estimated the five multi-item factors within a single confirmatory model, retaining the two residual covariances specified for parental support.

Table S5. Five-factor measurement model containing all multi-item constructs

Panel A. Factors, loadings and variance extracted				
**Factor**	**Items**	**Loading range**	**AVE**	**√AVE**
Physical somatic	4	0.665–0.750	0.501	0.708
Psychological somatic	4	0.586–0.890	0.646	0.804
School alienation	3	0.740–0.810	0.585	0.765
Parental support	6	0.735–0.886	0.649	0.806
Peer victimization	6	0.783–0.865	0.697	0.835
Panel B. Factor correlations				
	**Physical**	**Psychological**	**School alienation**	**Parental support**
Psychological somatic	0.732	—		
School alienation	0.171	0.255	—	
Parental support	−0.034	−0.126	−0.431	—
Peer victimization	0.391	0.466	0.439	−0.115

Model fit: $\chi^2(218) = 1132.74$; CFI = 0.988; TLI = 0.986; RMSEA = 0.034; SRMR = 0.044. AVE, average variance extracted. Estimated with the WLSMV estimator and delta parameterization, with the two residual covariances of the parental support model retained. School alienation is reported in the reverse-scored orientation used throughout the paper, so that higher values indicate greater alienation. All correlations are significant at $p < 0.001$ except that between physical somatic burden and parental support ($p = 0.127$). N =3523.

The joint model fits well, and the loadings and factor correlations closely reproduce those obtained from the separate models, indicating that the construct-by-construct specification does not distort the measurement structure. Within this model the school alienation factor is embedded in an over-identified structure, so that its fit can be evaluated, and the model as a whole meets conventional criteria. The correlations among the psychosocial constructs behave as expected, with peer victimization and school alienation correlated positively and parental support correlated negatively with both. The correlation between the two somatic factors (0.732) is essentially unchanged from the value obtained in the somatic two-factor model reported in the main text.

Two of the measurement decisions taken in this section were guided by the data: the removal of items that did not load on the intended factor, and the freeing of two residual covariances in the parental support model. Both were taken in the same sample used for the substantive analyses, and both are therefore exploratory rather than confirmatory. To establish whether they reflect stable features of the data or sample-specific noise, we divided the analytic sample at random into two halves of equal size and repeated the measurement analyses independently in each.

Table S6. Replication of the measurement decisions in two random halves of the analytic sample

Residual covariance	Full sample	Half A (n = 1762)	Half B (n = 1761)
Panel A. Modification index (expected parameter change), rank, for the two freed residual covariances			
Helps me as much as I need ↔ Shows they care about me	76.56 (+0.095), 1st	24.82 (+0.076), 2nd	55.08 (+0.115), 1st
Tries to understand me ↔ Encourages my own decisions	56.81 (+0.081), 2nd	24.78 (+0.075), 3rd	31.36 (+0.086), 2nd
Largest competing pair not freed	*54.01 (−0.088), 3rd*	*35.18 (−0.100), 1st*	*20.30 (−0.076), 4th*
Panel B. Somatic measurement model in each half		**Half A**	**Half B**
One-factor model, CFI / RMSEA / SRMR		0.954 / 0.129 / 0.078	0.955 / 0.136 / 0.078
Two-factor model, CFI / RMSEA / SRMR		0.986 / 0.072 / 0.048	0.987 / 0.076 / 0.047
**Scaled $\Delta\chi^2$ (Δdf = 1)**		**196.01, p < 0.001**	**233.53, p < 0.001**
Factor correlation		0.728	0.739

Predictor	Physical, A / B	Psychological, A / B
Panel C. Odds ratios estimated independently in each half		
Peer victimization	1.372 / 1.480	1.901 / 1.804
School alienation	1.143 / 1.099	1.179 / 1.166
Parental support	1.030 / 0.984	0.935 / 0.840
BMI z-score	0.953 / 0.962	0.949 / 1.032
Healthy diet	0.825 / 0.853	0.874 / 0.845
Unhealthy diet	1.011 / 0.993	1.066 / 1.021
Physical activity	1.080 / 1.045	1.150 / 1.082
Chronic illness	1.855 / 1.387	1.559 / 1.210
Organized sport	1.611 / 1.232	1.455 / 1.681
Income strain	1.051 / 0.929	1.064 / 1.030
Housing problems	0.988 / 1.119	0.903 / 1.030
Age	1.245 / 1.278	1.269 / 1.144
Sex (female)	2.399 / 2.307	1.942 / 1.698

The sample was split at random with a fixed seed. Panel A reports the modification index for each residual covariance in the unmodified parental support model, with the expected parameter change in parentheses and the rank of that index among all candidate modifications. Panel C gives odds ratios from the survey-weighted ordinal models estimated separately within each half.

Three findings emerge. First, the two residual covariances freed in the parental support model rank among the three largest modification indices in the full sample and in each half, and their expected parameter change is positive throughout, which is the pattern expected of items that share content. The competing pair with the largest index in half A carried a negative expected parameter change; a negative residual covariance indicates that the model over-predicts the association between two items rather than that the items overlap in content, and freeing it would not have been theoretically defensible. Second, the advantage of the two-factor somatic model over the one-factor model replicates in both halves, and the factor correlation is stable across them. Third, the odds ratios estimated independently in each half reproduce the substantive pattern; the wider variation for chronic illness and organized sport reflects the smaller number of exposed adolescents once the sample is halved.

We nonetheless retain the description of these measurement decisions as exploratory. Replication within halves of the same sample establishes that the decisions are not artifacts of sampling noise, but it is not equivalent to replication in an independent sample, and the item selection remains a decision taken with sight of the data.

Sensitivity of the measurement models to missing data

Because the analytic chain proceeds on a single complete-case sample, we verified that this case base does not distort the measurement structure on which all subsequent stages depend. Each of the four measurement models was re-estimated on the full sample (4072 adolescents) using maximum likelihood with full-information maximum likelihood (FIML) handling of missing data, treating the items as continuous. To isolate the effect of the missing-data treatment, the estimator was held constant across the comparison, so that the only difference between the two fits was listwise deletion versus FIML; this design separates the consequence of including partially observed cases from the unrelated question of the estimator. The two solutions were effectively indistinguishable for every construct. For the somatic, parental support, and peer victimization factors, whose items were essentially complete, the complete-case and FIML samples were identical (n = 4072) and every standardized loading was unchanged to three decimal places. The decisive comparison was the school alienation factor, on which missingness was

concentrated: FIML recovered 81 additional cases (n = 3530 under listwise deletion versus 3611 under FIML), yet the standardized loadings moved by no more than 0.001 (0.581 to 0.582, 0.658 to 0.658, and 0.770 to 0.769). Across all four models, the maximum change in any standardized loading was 0.001, the factor correlation between the two somatic dimensions was unchanged (r = 0.677), and global fit was identical to the second decimal place (for example, for the somatic two-factor model, CFI = 0.972, TLI = 0.958, and RMSEA = 0.062 under both approaches, with the standardized root mean square residual differing only trivially, 0.041 versus 0.037). Including the partially observed cases through FIML therefore left the measurement structure unchanged, supporting the use of the complete-case analytic sample for the full analytic chain.

Bifactor model as an additional candidate structure

We fitted a bifactor model as a third candidate structure for the eight HBSC-SCL items, alongside the one-factor and two-factor models (Table S7). In the bifactor model, every item loads on a general somatic factor and, simultaneously, on an orthogonal specific factor for its own cluster, so that the specific factors capture what the items in a cluster share after the general factor has been partialled out. The model converged with no improper estimates and no Heywood cases. In keeping with the other latent-structure analyses in this study, the measurement models in Panels A and B were estimated on the unweighted analytic sample, and applying the sampling weights left every fit index and bifactor index essentially unchanged (ωH = 0.79 in both cases). We do not select among the models on fit alone. A bifactor model is more flexible than its competitors and will almost always fit better, so the decision rests on the loadings and on the bifactor indices rather than on the fit statistics (Dunn & McCray, 2020).

As expected, the bifactor model fits best on every index (Table S7, Panel A). Fit alone, however, does not settle the comparison. The general factor is strong and uniform (Table S7, Panel B). Every item loads on it between 0.53 and 0.74, and the general factor accounts for 72% of the common variance (ECV = 0.724) and 79% of the reliable variance in the total score (ωH = 0.785). The specific factors are weaker. Once the general factor is removed, the physical and psychological subscale scores retain very little reliable unique variance (ωHS = 0.183 for the physical and 0.210 for the psychological subscale). Difficulty sleeping in the psychological cluster (specific loading 0.060, p = .31) and, to a lesser degree, feeling dizzy in the physical cluster carry essentially no cluster-specific information, with item-level ECV values approaching 1.0. By these criteria, the eight-item checklist is dominated by a single general dimension, and the physical and psychological subscales should not be interpreted as independent or pure domain-specific measures, but as strongly overlapping manifestations of a dominant general symptom burden (Reise et al., 2013; Rodriguez et al., 2016).

This pattern qualifies rather than overturns the two-factor result. The distinction between physical and psychological complaints is real, but it is expressed at the latent level and, above all, in the external relations of the two dimensions, rather than in the reliable variance of the raw subscale scores. The bifactor MIMIC model, estimated with the sampling weights applied in line with the other structural regression models, makes the point directly (Table S7, Panel C). When the general factor and the two specific factors are regressed simultaneously on the 13 predictors (CFI = 0.994, RMSEA = 0.021, no Heywood cases), the specific factors do not relate uniformly to the predictors even with the shared core removed. The hypothesis of equal specific-factor paths is rejected, $\chi^2(13) = 69.04$, $p = 1.2 \times 10^{-9}$, as is the restriction on the four core paths, $\chi^2(4) = 36.22$, $p = 2.6 \times 10^{-7}$. Female sex is associated far more strongly with the physical specific factor (0.800) than with the psychological one (0.418), a difference of 0.382 ($p < .001$) that persists after the general factor is removed. Chronic illness and parental support show the same pattern of specific-factor separation. This provides an additional sensitivity analysis, showing that the specific-factor paths are not uniformly equal once the general factor is accounted for. Given the limited reliability and construct replicability of the specific factors, however, these residual-factor coefficients are interpreted cautiously and are not treated as stronger evidence than the correlated two-factor model.

Table S7. Bifactor model of the eight HBSC-SCL somatic items: model comparison, standardized loadings with bifactor indices, and bifactor MIMIC regressions

Panel A. Fit of the one-factor, two-factor, and bifactor models for the eight HBSC-SCL items (n = 3523)

Model	χ^2 (*df*)	CFI	RMSEA	SRMR
One-factor	1085.3 (20)	0.956	0.123	0.078
Two-factor (correlated)	345.4 (19)	0.986	0.070	0.048
Bifactor	69.8 (12)	0.998	0.037	0.019

Panel B. Standardized loadings on the general and specific factors, item-level explained common variance (I-ECV), and bifactor statistical indices

Item	General	Specific	Specific *p*	I-ECV
Physical cluster				
Headache	0.643	0.427	< .001	0.694
Stomachache	0.532	0.529	< .001	0.503
Backache	0.633	0.285	< .001	0.831
Feeling dizzy	0.663	0.120	.059	0.957
Psychological cluster				
Feeling low	0.744	0.334	< .001	0.832
Irritability	0.714	0.547	< .001	0.630
Feeling nervous	0.696	0.571	< .001	0.598
Difficulty sleeping	0.623	0.060	.31	0.999
Mean |loading|	0.656	0.345 / 0.366		
Bifactor statistical indices				
Explained common variance, general factor (ECV)				0.724
Percentage of uncontaminated correlations (PUC)				0.571
Coefficient omega, total score (ω)				0.907
Omega hierarchical, general factor (ωH)				0.785
Omega hierarchical subscale, physical (ωHS)				0.183
Omega hierarchical subscale, psychological (ωHS)				0.210
Construct replicability H, general factor				0.865
Construct replicability H, physical specific				0.419
Construct replicability H, psychological specific				0.509

Panel C. Bifactor MIMIC model: standardized regressions of the general factor and the two specific factors on the 13 predictors

Predictor	General	Phys. spec.	Psych. spec.	Spec. diff.	*p*
Peer victimization	0.298***	−0.061	0.042	−0.103	.020
School alienation	0.100**	−0.004	0.084*	−0.088	.030
Parental support	−0.129***	0.141***	0.008	0.134	.001
BMI z-score	−0.037	0.010	0.056	−0.046	.225
Healthy diet	−0.132***	−0.005	0.046	−0.051	.192
Unhealthy diet	−0.019	0.038	0.052	−0.014	.707
Physical activity	0.031	−0.005	0.059	−0.065	.113
Income strain	0.052	−0.043	−0.015	−0.028	.465
Housing problems	0.013	0.044	−0.021	0.065	.093
Age	0.196***	−0.030	−0.015	−0.014	.722
Sex (female)	0.230***	0.800***	0.418***	0.382	< .001
Chronic illness	0.301**	0.172	−0.120	0.292	.011
Organized sport	0.270***	−0.082	0.101	−0.183	.070

All models were estimated with the WLSMV estimator and the delta parameterization, treating the five ordinal categories as ordered; fit statistics in Panel A are scaled. In Panel B, loadings are the fully standardized solution, with the specific factors orthogonal to the general factor and to each other; I-ECV is the proportion of an item's common variance attributable to the general factor. In Panel C, coefficients are standardized; the specific-factor difference is the physical-specific minus the psychological-specific path. ECV, explained common variance; PUC, percentage of uncontaminated correlations; ω, coefficient omega; ωH, omega hierarchical; ωHS, omega hierarchical subscale; H, construct replicability. *** p < .001, ** p < .01, * p < .05.

Two features of the MIMIC results require the caution appropriate to specific factors. Peer victimization loads clearly on the general factor (0.298, $p < .001$), but its specific paths are individually non-significant, and its cleaner association with the psychological dimension in the correlated-factor models largely reflects an elevation of the general level. In addition, because the specific factors are residuals, their coefficients are not interpreted on the same footing as the general-factor coefficients. The positive physical-specific path for parental support, set against its negative general-factor path, is therefore not a substantive reversal (Dunn & McCray, 2020). Neither feature alters the central finding, which rests on the general factor and on the external separation of the specific factors.

Section S3: Multiple Correspondence Analysis of the Psychosocial Microsystems

The three psychosocial constructs (peer victimization, school alienation, and parental support) were each summarized as ordered categories before being entered into a multiple correspondence analysis (MCA). Because the school alienation and parental support distributions were strongly peaked at their modal values and peer victimization was concentrated at zero (74.4% reporting no exposure), we used substantively meaningful cut points rather than equal-sized groups: peer victimization was coded as none, low, or high; school alienation and parental support as low, moderate, or high relative to their central values (Table S8). The MCA was estimated on the unweighted analytic sample, consistent with our treatment of the other latent-structure analyses.

Table S8. Category distributions of the three psychosocial domains entered into the multiple correspondence analysis

Domain	Category	n	%
Peer victimization	None	2622	74.4
	Low	261	7.4
	High	640	18.2
School alienation	Low	927	26.3
	Moderate	1769	50.2
	High	827	23.5
Parental support	Low	1048	29.7
	Moderate	1500	42.6
	High	975	27.7

The first dimension accounted for 22.8% of the raw inertia; because the raw inertia of MCA is known to be deflated by the indicator coding, we also computed Benzécri-adjusted inertia, under which the first dimension explained 62.9% and the first two dimensions 99.9% of the adjusted inertia. The first dimension ordered the categories along a single psychosocial adversity gradient, and the second dimension reflected a contrast between middle and extreme categories characteristic of the arch (Guttman) effect rather than a substantive second axis. Category coordinates, contributions, and squared correlations with each dimension are reported in Table S9.

Table S9. Category coordinates, contributions, and squared correlations from the multiple correspondence analysis

	Category	Dim 1	Dim 2	Contrib. 1 (%)	Contrib. 2 (%)	η^2 (Dim 1 / 2)
Peer victimization	None	−0.270	−0.287	3.97	4.77	0.26 / 0.26
	Low	0.093	0.434	0.05	1.08	
	High	1.070	0.998	15.16	14.09	
School alienation	Low	−1.179	0.703	26.68	10.14	0.63 / 0.54
	Moderate	0.135	−0.728	0.67	20.72	
	High	1.033	0.769	18.29	10.80	
Parental support	Low	0.785	0.401	13.37	3.72	0.48 / 0.49
	Moderate	0.120	−0.797	0.45	21.05	
	High	−1.029	0.795	21.37	13.63	

Dim 1 and Dim 2 are the first two MCA dimensions. Contrib., contribution of the category to the dimension inertia; η^2, squared correlation of the domain with the dimension (reported once per domain). Higher Dimension 1 values indicate greater psychosocial adversity.

We carried out two robustness checks on the first dimension. First, re-estimating the MCA with a coarser binary coding of all three domains (no versus any victimization, and median splits of school alienation and parental support) produced a first-dimension score that correlated strongly with the original (Spearman $r = 0.83$), indicating that the adversity gradient was not an artifact of the specific cut points. Second, the first-dimension score correlated highly with a simple standardized mean of the three domains (Pearson $r = 0.83$; Spearman $r = 0.92$), confirming that the dimension can be interpreted straightforwardly as an overall psychosocial adversity score. Together these checks support the use of the first dimension as a composite adversity score in the sensitivity analysis, while the

unequal loadings of the three domains on this dimension (Table S9) motivated their inclusion as separate predictors in the main models.

Table S10. Ordinal trend between each psychosocial domain and somatic symptom category

Domain (category)	Low somatic %	Moderate %	High somatic %	*Z, p*
Peer victimization				
None	46.8	30.5	22.7	***Z = 17.33, p < 0.001***
Low	23.0	38.7	38.3	
High	18.0	27.5	54.5	
School alienation				
Low	45.5	31.5	23.0	***Z = 10.11, p < 0.001***
Moderate	42.7	31.4	25.8	
High	27.1	27.6	45.3	
Parental support				
Low	32.0	28.7	39.3	***Z = −4.64, p < 0.001***
Moderate	46.7	29.7	23.6	
High	37.6	33.7	28.6	
Composite adversity score, tertiles				
Lowest adversity	44.5	33.5	22.0	***Z = 10.32, p < 0.001***
Middle	44.5	31.4	24.0	
Highest adversity	30.3	26.7	43.0	

Row percentages show the distribution of somatic symptom category within each level of the domain. Z and p are from the linear-by-linear association test for ordinal trend. All analyses are unweighted and descriptive; weighted multivariable models are reported in the main text.

As a preliminary, unweighted check on the association between the psychosocial microsystems and somatic burden, we tested the ordinal trend between each domain and the three-level somatic symptom category using the linear-by-linear association test. Each domain showed a significant trend: greater peer victimization ($Z = 17.33$, $p < 0.001$) and greater school alienation ($Z = 10.11$, $p < 0.001$) were associated with higher somatic burden, and greater parental support with lower somatic burden ($Z = -4.64$, $p < 0.001$), although the gradient for parental support was weaker and not strictly monotonic. The composite adversity score showed the same pattern, with the proportion in the high somatic category rising from 22.0% in the lowest adversity tertile to 43.0% in the highest ($Z = 10.32$, $p < 0.001$). These descriptive trends are consistent with the survey-weighted models reported in the main text and are summarized in Table S10.

Section S4: Latent Profile Analysis of Lifestyle and Socioeconomic Indicators

Six lifestyle and socioeconomic indicators were evaluated for a possible latent profile structure: BMI z-score, healthy diet, unhealthy diet, physical activity (days per week), household income strain, and housing problems. All indicators were z-standardized prior to analysis. We first examined the pairwise associations among them. With the single exception of the correlation between household income strain and housing problems ($r = 0.285$), every correlation was negligible ($|r| < 0.11$), so that the indicators were largely orthogonal to one another (Figure S1). The corresponding Spearman coefficients were closely comparable (income strain with housing problems, $\rho = 0.274$; all remaining $|\rho| < 0.13$), confirming that the near-independence of the indicators was not an artifact of the correlation metric.

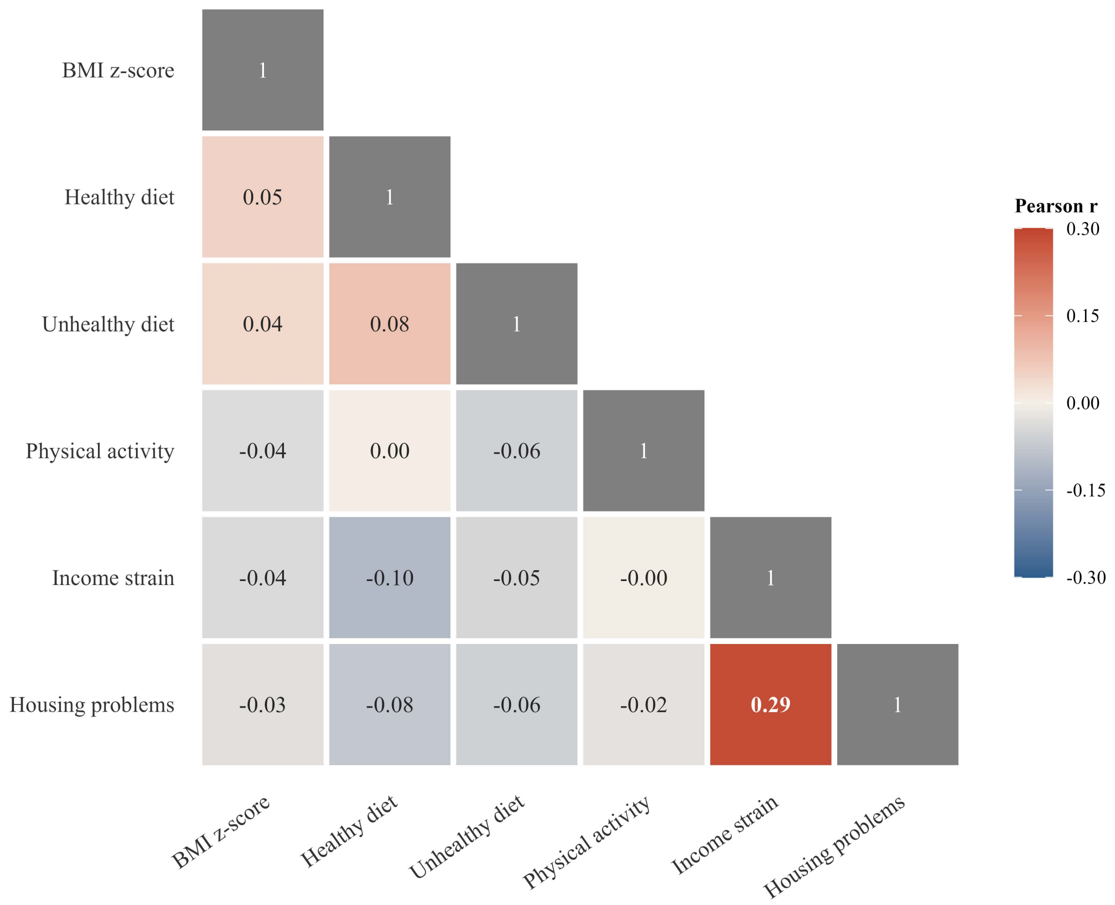

Figure S1. Pearson correlations among the six lifestyle and socioeconomic indicators

Latent profile analysis was then carried out in tidyLPA (Rosenberg et al., 2018) for one- to six-profile solutions using Model 1, which assumes equal variances and zero covariances across indicators (Nylund-Gibson & Choi, 2018; Spurk et al., 2020). The fit indices are reported in Table S11. The BIC decreased monotonically as profiles were added but did not reach a clear minimum, and entropy remained below 0.70 for every solution other than the two-profile model, indicating poorly separated classes. A more flexible specification permitting varying variances failed to converge. This pattern is more consistent with the arbitrary partitioning of a set of orthogonal continuous indicators than with the recovery of a genuine latent profile structure (Table S11).

Table S11. Fit indices for latent profile models fitted to the lifestyle and socioeconomic indicators

Model: equal variances, zero covariances						
**Classes**	**LogLik**	**AIC**	**BIC**	**saBIC**	**Entropy**	**BLRT p**
1	−29,991	60,005	60,079	60,041	1.000	—
2	−29,288	58,614	58,731	58,670	0.867	0.010
3	−29,217	58,486	58,647	58,564	0.634	0.010
4	−28,208	56,482	56,686	56,581	0.659	0.010
5	−28,079	56,237	56,484	56,357	0.672	0.010
6	−27,862	55,818	56,108	55,959	0.698	0.010

Model specifies equal variances and zero covariances across indicators, the most parsimonious LPA specification. LogLik, log-likelihood; AIC, Akaike information criterion; BIC, Bayesian information criterion; saBIC, sample-size adjusted BIC; BLRT, bootstrap likelihood ratio test comparing the k-profile model against the (k−1)-profile model. A more flexible specification with varying variances did not converge. n = 3523.

Considered jointly, the correlation structure and the latent profile fit indices did not support an interpretable profile solution. We therefore did not reduce the lifestyle and socioeconomic indicators to a single profile variable; instead, each was entered as a separate predictor in the main models. This decision parallels the logic adopted for the psychosocial microsystems in the multiple correspondence analysis, in which each system layer was treated as carrying independent information. The two binary indicators, participation in organized sport and chronic illness, were excluded from the latent profile analysis because they violate the Gaussian assumption underlying the model; these were instead modeled directly as covariates.

Section S5: Ordinal Regression and Robustness of the Outcome Specification

The two somatic dimensions identified in the measurement model, physical and psychological somatic burden, were modeled as separate ordinal outcomes, each collapsed into three categories (low, moderate, high). For each outcome we fitted a survey-weighted ordinal logistic regression under the proportional odds assumption, applying the final person-level sampling weight (FAKTOR_FERT_1317) through a survey design object. Thirteen predictors were entered simultaneously: the three psychosocial microsystem variables (peer victimization, school alienation, and parental support) as separate terms, the six lifestyle and socioeconomic indicators, and age, sex, chronic illness, and organized sport participation. Multicollinearity was not a concern, with all variance inflation factors below 1.2. The full coefficients for both models are reported in Table S12, which provides the numerical detail underlying the forest plot summarized in the main text (Figure 2).

Table S12. Ordinal logistic regression coefficients for physical and psychological somatic burden

	Physical somatic burden			Psychological somatic burden		
**Predictor**	**OR**	**95% CI**	**p**	**OR**	**95% CI**	**p**
Psychosocial microsystems						
Peer victimization	1.43	1.27, 1.61	<0.001	1.84	1.60, 2.11	<0.001
School alienation	1.12	1.04, 1.21	0.001	1.18	1.09, 1.27	<0.001
Parental support	1.00	0.93, 1.07	0.969	0.88	0.82, 0.95	0.001
Lifestyle and health						
BMI z-score	0.96	0.90, 1.02	0.209	0.99	0.92, 1.06	0.740
Healthy diet	0.84	0.78, 0.90	<0.001	0.86	0.80, 0.92	<0.001
Unhealthy diet	1.00	0.94, 1.07	0.909	1.04	0.97, 1.11	0.246
Physical activity	1.06	0.99, 1.14	0.087	1.11	1.04, 1.19	0.002
Chronic illness	1.61	1.28, 2.03	<0.001	1.37	1.08, 1.74	0.008
Organized sport	1.42	1.20, 1.68	<0.001	1.57	1.33, 1.86	<0.001
Socioeconomic context						
Income strain	0.99	0.92, 1.06	0.741	1.04	0.97, 1.12	0.218
Housing problems	1.05	0.98, 1.13	0.160	0.97	0.90, 1.04	0.381
Sociodemographic						
Age	1.26	1.18, 1.34	<0.001	1.21	1.13, 1.29	<0.001
Sex (female)	2.35	2.04, 2.71	<0.001	1.82	1.58, 2.09	<0.001

Survey-weighted ordinal logistic regression (proportional odds); both outcomes were coded low/moderate/high. OR, odds ratio; CI, confidence interval. All continuous predictors were standardized, so their odds ratios are expressed per standard deviation increase and are directly comparable across predictors; sex, chronic illness, and organized sport are binary, with reference categories male, no chronic illness, and no organized sport participation. p-values were derived from the Wald z statistic; values below 0.001 are reported as <0.001. n = 3523.

The proportional odds assumption was assessed by comparing the predictor coefficients across the two cumulative binary thresholds defined by each outcome (≥ moderate and = high), following the graphical approach of Harrell (Harrell, 2015). Departures from proportionality were small for the physical (mean absolute difference in log-odds 0.06) and overall (0.09) outcomes. For the psychological outcome (0.10), all predictors showed small departures except peer victimization, whose effect was somewhat stronger at the lower threshold. We retained the proportional odds model because the assumption held for every other predictor and because the direction and statistical significance of each effect were consistent across the two thresholds.

As a sensitivity analysis, we replaced the three separate psychosocial variables with a single multiple correspondence analysis-based adversity score (the first dimension derived in Section S3). This composite axis was positive and significant for both outcomes (physical OR 1.53, 95% CI 1.38, 1.69; psychological OR 1.90, 95% CI 1.72, 2.10), and the stronger association with psychological somatic burden mirrored the pattern observed in the main model. The psychosocial effect was therefore robust to whether it was modeled as separate variables or as a single composite axis. Finally, we examined possible nonlinear relationships for body mass index and age using survey-weighted generalized additive models (mgcv; thin-plate regression splines) (Wood, 2017). For both outcomes the smooth term for BMI had an effective degrees of freedom of approximately one and was not significant (physical edf 1.00, p = 0.47; psychological edf 1.00, p = 0.80), indicating no nonlinear, and in particular

no U-shaped, relationship between BMI and somatic burden, contrary to the pattern previously reported in clinical samples (S. Chen et al., 2024). The association with age was linear and significant (edf 1.00, $p < 0.001$). These results support the linearity assumption underlying the main ordinal models.

The primary models treat each somatic dimension as an ordered three-level outcome. Both subscales are bounded, strongly right-skewed and zero-inflated, with 31.8% of adolescents reporting no physical symptom and 27.7% no psychological symptom, and the ordinal specification was chosen to accommodate these features while preserving a common metric across the two dimensions. Categorization nevertheless discards information, and the cut points used for the subscales differ from the tertiles used for the total score, because the concentration of scores at zero makes an equal-sized tertile split of the subscales impossible. Since the comparative argument of the paper rests on these outcomes, we re-estimated the associations under five further specifications: a four-level outcome that retains zero as its own category and divides the non-zero scores into tertiles; a hurdle decomposition, in which a logistic model estimates the presence of any symptom and a gamma model with a log link estimates the severity of the score among adolescents reporting at least one symptom; a survey-weighted linear model of the standardized continuous subscale score; and a repetition of the primary ordinal model in which the mean-score psychosocial predictors are replaced by latent factor scores from the measurement models.

Table S13. Associations with each somatic dimension under the primary and five alternative specifications of the outcome and of the psychosocial predictors

Predictor	Three-level (primary)	Four-level, non-zero tertiles	Hurdle: any symptom	Hurdle: severity	Continuous score	Latent factor-score predictors
Panel A. Physical somatic burden						
Peer victimization	1.429	1.390	1.357	1.120	0.184	1.440
School alienation	1.121	1.130	1.086	1.051	0.060	1.116
Parental support	1.001	0.989	1.020	0.967	−0.024	1.013
BMI z-score	0.958	0.950	0.956	0.998	−0.012	0.957
Healthy diet	0.842	0.841	0.872	0.947	−0.082	0.841
Unhealthy diet	1.004	1.023	0.994	1.006	0.006	1.006
Physical activity	1.061	1.064	1.104	0.996	0.014	1.062
Chronic illness	1.612	1.599	1.484	1.233	0.314	1.613
Organized sport	1.421	1.413	1.392	1.076	0.139	1.420
Income strain	0.988	1.014	0.935	1.033	0.018	0.989
Housing problems	1.054	1.045	1.055	1.020	0.025	1.055
Age	1.259	1.255	1.240	1.066	0.115	1.259
Sex (female)	2.351	2.462	2.402	1.286	0.443	2.353
Panel B. Psychological somatic burden						
Peer victimization	1.840	1.664	2.514	1.110	0.245	1.862
School alienation	1.177	1.197	1.107	1.081	0.109	1.162
Parental support	0.883	0.873	0.973	0.916	−0.091	0.893
BMI z-score	0.988	0.984	0.984	1.006	−0.004	0.988
Healthy diet	0.860	0.873	0.845	0.975	−0.063	0.859
Unhealthy diet	1.041	1.034	1.040	1.005	0.017	1.043
Physical activity	1.114	1.113	1.198	1.009	0.043	1.116
Chronic illness	1.374	1.335	1.273	1.118	0.160	1.368
Organized sport	1.573	1.516	1.486	1.133	0.224	1.578
Income strain	1.044	1.061	1.053	1.018	0.033	1.045
Housing problems	0.968	0.976	0.953	1.016	−0.005	0.972
Age	1.205	1.245	1.204	1.081	0.119	1.204
Sex (female)	1.820	1.903	1.708	1.234	0.321	1.829

All models are survey-weighted and adjusted for the same 13 predictors. Columns 1 to 4 and column 6 report odds ratios or exponentiated coefficients; column 5 reports a linear coefficient for the standardized continuous subscale. The hurdle columns decompose the outcome into the presence of any symptom and, conditional on presence, its severity.

The estimates are stable across specifications. Among the directly comparable models, replacing the mean-score psychosocial predictors with latent factor scores changed every odds ratio by no more than 0.03. The four-level categorization preserved the direction and the substantive ranking of the key associations, although some magnitudes shifted; peer victimization, for instance, moved from 1.84 to 1.66 on the psychological dimension.

Across the hurdle, continuous and latent-variable specifications the central differential pattern was reproduced: peer victimization and parental support remained more closely related to psychological burden, and female sex and chronic illness to physical burden. Estimates that were already close to the null occasionally crossed 1.0, but no substantive conclusion changed. The hurdle and continuous models are on different scales and are not numerically comparable with the ordinal models; what they establish is that the direction, the relative ordering, and the differential attachment of predictors to the two dimensions are preserved. The differential pattern is preserved throughout: peer victimization and parental support attach more strongly to psychological burden, and female sex and chronic illness to physical burden, under every specification. The hurdle decomposition adds an interpretable detail. Peer victimization is associated principally with the presence of any psychological symptom (odds ratio 2.51) rather than with the severity of symptoms once present (1.11), whereas its association with the presence of physical symptoms is considerably smaller (1.36). Female sex, by contrast, is associated with both the presence and the severity of physical symptoms (2.40 and 1.29). The distinction between whether symptoms occur at all and how severe they become is not one the ordinal models can express, and it is offered here as description rather than as a further claim.

Stratum and primary sampling unit identifiers were not released with the microdata, and the survey design could therefore be specified with sampling weights alone. If the sample was clustered, as is usual for a national household survey, the standard errors obtained under this specification are underestimated and the corresponding p-values are anticonservative. The design effect cannot be estimated from the released data. We therefore examined how far each association would survive a range of plausible design effects, by multiplying each standard error by the square root of an assumed design effect and recomputing the p-value.

Table S14. p-values for each association under progressively inflated standard errors

Predictor	Odds ratio	Assumed design effect			
		1.0	1.5	2.0	3.0
Panel A. Physical somatic burden					
Peer victimization	1.429	<0.001	<0.001	<0.001	<0.001
School alienation	1.121	0.003	0.014	0.033	0.081
Parental support	1.001	0.970	0.975	0.979	0.983
BMI z-score	0.958	0.209	0.305	0.375	0.469
Healthy diet	0.842	<0.001	<0.001	0.001	0.006
Unhealthy diet	1.004	0.909	0.925	0.935	0.947
Physical activity	1.061	0.087	0.163	0.227	0.324
Chronic illness	1.612	<0.001	0.001	0.004	0.020
Organized sport	1.421	<0.001	<0.001	0.004	0.018
Income strain	0.988	0.741	0.788	0.816	0.849
Housing problems	1.054	0.160	0.251	0.320	0.417
Age	1.259	<0.001	<0.001	<0.001	<0.001
Sex (female)	2.351	<0.001	<0.001	<0.001	<0.001
Panel B. Psychological somatic burden					
Peer victimization	1.840	<0.001	<0.001	<0.001	<0.001
School alienation	1.177	<0.001	0.001	0.004	0.019
Parental support	0.883	0.001	0.009	0.023	0.064
BMI z-score	0.988	0.740	0.786	0.814	0.848
Healthy diet	0.860	<0.001	<0.001	0.002	0.011
Unhealthy diet	1.041	0.246	0.344	0.412	0.503
Physical activity	1.114	0.002	0.011	0.027	0.071
Chronic illness	1.374	0.008	0.031	0.062	0.128
Organized sport	1.573	<0.001	<0.001	<0.001	0.002
Income strain	1.044	0.219	0.315	0.384	0.477
Housing problems	0.968	0.381	0.474	0.536	0.613
Age	1.205	<0.001	<0.001	<0.001	0.002
Sex (female)	1.820	<0.001	<0.001	<0.001	<0.001

Each p-value is recomputed after multiplying the standard error from the survey-weighted ordinal model by the square root of the assumed design effect. A design effect of 1.0 corresponds to the analysis as reported.

The core associations are robust. Peer victimization, healthy diet, age, female sex and organized sport with both dimensions, and chronic illness with physical burden, remain significant at a design effect of 2, used here as an illustrative assumed value rather than as an estimate recoverable from the released data. Four associations are less secure: parental support, chronic illness and physical activity with psychological burden, and school alienation with physical burden, would not remain significant at a design effect of 3. These are also the associations that were smallest in magnitude, and they are interpreted with corresponding caution in the discussion. The same procedure is applied to the latent path-difference tests in Section S6 (Table S16).

Section S6: Latent-Variable Model and Formal Tests of Differential Association

The central empirical claim of this study is comparative: that the two somatic dimensions attach differently to the surrounding system, with peer victimization and parental support more closely tied to psychological burden and female sex and chronic illness more closely tied to physical burden. In the regression analysis reported in Section S5, that claim rests on the comparison of two separately estimated models. Such a comparison is informative but it is not a test. Two coefficients may differ in point estimate while their confidence intervals overlap; separately fitted models supply no covariance between the two estimates, so the precision of their difference cannot be obtained; and both estimates inherit the measurement error in the observed subscale scores, which attenuates them to a degree that need not be equal across dimensions.

We therefore re-estimated the associations within a single latent-variable model. The physical and psychological somatic factors, each defined by its four ordinal items, were regressed simultaneously on all thirteen predictors in a multiple-indicator multiple-cause model (Jöreskog & Goldberger, 1975), estimated with the WLSMV estimator, delta parameterization and sampling weights. The latent factors were scaled to unit variance and all continuous predictors were standardized, so that the coefficients for the two dimensions lie on a common scale and can be compared directly. Because both regressions are estimated within one model, the covariance between any pair of coefficients is available, and the difference between them can be tested by a Wald test of the corresponding equality constraint. The model addresses three concerns at once: it removes the categorization of the outcome, it corrects for measurement error in the outcome, and it converts the comparison of the two dimensions from a juxtaposition of point estimates into a formal within-sample test. Because the pattern being tested and the model testing it are estimated from the same data, it is a within-sample test of a pattern already described rather than independent confirmation of it, and establishing that would require external replication, ideally preregistered.

The model and its tests are reported in the main text (Table 3). Table S15 gives the fully standardized coefficients and examines whether the difference tests depend on the estimation scheme, comparing the primary weighted latent model with the same model estimated without sampling weights and with a survey-weighted stacked linear model of the two standardized continuous subscales fitted to thirty imputed datasets and pooled by Rubin's rules.

Table S15. Standardized coefficients and the physical-minus-psychological difference under three estimation schemes

Predictor	Standardized β		Δ (physical − psychological), estimate (p)		
	Physical	Psychological	Weighted latent	Unweighted latent	Imputed continuous
**Peer victimization**	**0.195**	**0.253**	**−0.062 (0.003)**	**−0.043 (0.019)**	**−0.068 (0.002)**
School alienation	0.074	0.117	−0.047 (0.057)	−0.062 (0.003)	−0.047 (0.033)
**Parental support**	**−0.024**	**−0.094**	**0.076 (<0.001)**	**0.079 (<0.001)**	**0.058 (<0.001)**
BMI z-score	−0.018	−0.002	−0.018 (0.441)	−0.019 (0.375)	0.008 (0.641)
Healthy diet	−0.109	−0.071	−0.042 (0.056)	−0.038 (0.067)	−0.020 (0.252)
Unhealthy diet	0.006	0.012	−0.007 (0.747)	0.013 (0.509)	−0.022 (0.165)
Physical activity	0.025	0.051	−0.028 (0.242)	−0.040 (0.075)	−0.015 (0.367)
**Chronic illness**	**0.102**	**0.048**	**0.193 (0.003)**	**0.169 (0.005)**	**0.136 (0.018)**
Organized sport	0.071	0.102	−0.083 (0.150)	−0.106 (0.045)	−0.099 (0.023)
Income strain	0.017	0.031	−0.016 (0.483)	−0.023 (0.287)	−0.006 (0.727)
Housing problems	0.038	−0.005	0.047 (0.042)	0.060 (0.005)	0.023 (0.176)
Age	0.138	0.137	0.002 (0.938)	0.012 (0.576)	−0.003 (0.869)
**Sex (female)**	**0.290**	**0.191**	**0.218 (<0.001)**	**0.208 (<0.001)**	**0.112 (<0.001)**
Omnibus Wald tests of equality across the two dimensions					
All 13 paths	Weighted: $\chi^2(13) = 83.74$, $p < 0.001$		Unweighted: $\chi^2(13) = 93.80$, $p < 0.001$		
Psychosocial microsystems	Weighted: $\chi^2(3) = 31.86$, $p < 0.001$		Unweighted: $\chi^2(3) = 38.50$, $p < 0.001$		
Biological–demographic	Weighted: $\chi^2(2) = 31.34$, $p < 0.001$		Unweighted: $\chi^2(2) = 30.01$, $p < 0.001$		
Four core paths	Weighted: $\chi^2(4) = 54.14$, $p < 0.001$		Peer victimization, parental support, sex, chronic illness		

The difference is defined throughout as the physical coefficient minus the psychological coefficient, so a positive value indicates a stronger association with physical burden. Standardized coefficients are from the weighted latent model. Rows in bold are the four paths that differ significantly in the primary model and in every sensitivity specification.

The omnibus constraint that all thirteen paths are equal across the two dimensions is rejected decisively, as are the constraints applied separately to the three psychosocial paths and to the two biological and demographic paths.

Four paths differ significantly between dimensions in the primary model and in every sensitivity specification: peer victimization and parental support are more strongly associated with psychological burden, and female sex and chronic illness with physical burden. Tested jointly, these four constraints are rejected at $\chi^2(4) = 54.14$, $p < 0.001$. The differential pattern reported in the main text is therefore not an artifact of comparing two separately fitted models, nor of categorizing the outcomes, nor of measurement error in the subscale scores.

The bidirectionality of the pattern is what argues against reading it purely as an artifact of item content. It may reasonably be objected that the four psychological items concern low mood, irritability, nervousness and difficulty sleeping, that these overlap in content with internalizing symptoms, and that an association between peer victimization and this dimension may therefore partly reflect that overlap rather than a substantive difference between two forms of somatic burden. Content overlap of this kind, however, would be expected to produce a largely one-sided pattern: it would inflate the paths running from the psychosocial microsystems to the psychological dimension. It does not readily explain why chronic illness and female sex attach more strongly to the physical dimension, nor why peer victimization retains a clear and independent association with physical burden. The dissociation is unlikely to be wholly attributable to the content of the psychological items, although we cannot exclude, on cross-sectional data from a single informant, that content overlap contributes to its magnitude.

Two differences that reach significance in some specifications are not treated as robust. The difference for school alienation is significant in the unweighted latent model and in the imputed continuous model but not in the primary weighted model, and the difference for organized sport is significant in the same two sensitivity models but not in the primary one. A difference for housing problems reaches significance in the latent models although neither of its individual paths is significant, and it is not interpreted.

One further check concerns the sampling design rather than the model. Because stratum and primary sampling unit identifiers were not released with the microdata, the survey design underlying the latent model, like that underlying the ordinal regressions, could be specified with sampling weights alone, and any clustering in the sample leaves the standard errors of the path differences underestimated to an unknown degree. We therefore applied to the difference tests the same procedure used for the individual associations in Section S5, multiplying the standard error of each coefficient difference by the square root of an assumed design effect and recomputing the p-value, and dividing each omnibus Wald statistic by the same factor, under the assumption that unaccounted clustering inflates the coefficient covariance matrix uniformly (Table S16).

The four core differences are robust to moderate clustering. All four remained significant at an assumed design effect of 2; the differences for female sex and parental support remained significant at a design effect of 3, whereas those for peer victimization and chronic illness fell marginally short at that level ($p = 0.086$). The omnibus tests were more robust still, with critical design effects between 3.7 and 5.7, so the joint conclusion that the two dimensions attach differently to the system does not depend on the absence of clustering. Two secondary observations reinforce decisions already taken: the difference for housing problems, which is not interpreted, would not survive a design effect of 1.5, and the difference for school alienation, which is not treated as robust, is not significant even at a design effect of 1. As in Section S5, the assumed design effects are illustrative values rather than estimates recoverable from the released data.

Table S16. Sensitivity of the latent path-difference tests to the unobserved sampling design: p-values under progressively inflated standard errors

Predictor	Δβ (SE)	Assumed design effect				Critical design effect
		1.0	1.5	2.0	3.0	
Panel A. Differences between the physical and psychological paths (Δβ)						
**Peer victimization**	**−0.062 (0.021)**	**0.003**	**0.015**	**0.036**	**0.086**	**2.30**
School alienation	−0.047 (0.025)	0.056	0.119	0.177	0.270	—
**Parental support**	**0.076 (0.022)**	**<0.001**	**0.004**	**0.013**	**0.042**	**3.24**
BMI z-score	−0.018 (0.023)	0.441	0.530	0.586	0.657	—
Healthy diet	−0.042 (0.022)	0.056	0.119	0.177	0.270	—
Unhealthy diet	−0.007 (0.021)	0.749	0.794	0.821	0.853	—
Physical activity	−0.028 (0.024)	0.242	0.339	0.408	0.499	—
**Chronic illness**	**0.193 (0.065)**	**0.003**	**0.015**	**0.036**	**0.086**	**2.30**
Organized sport	−0.083 (0.058)	0.150	0.240	0.309	0.406	—
Income strain	−0.016 (0.022)	0.484	0.568	0.621	0.686	—
Housing problems	0.047 (0.023)	0.041	0.096	0.149	0.239	1.08
Age	0.002 (0.022)	0.936	0.948	0.955	0.963	—
**Sex (female)**	**0.218 (0.047)**	**<0.001**	**<0.001**	**0.001**	**0.007**	**5.60**
Panel B. Omnibus Wald tests of the equality of paths across the two dimensions (χ^2 (df))						
All 13 paths	83.74 (13)	<0.001	<0.001	<0.001	0.009	3.74
Psychosocial microsystems	31.86 (3)	<0.001	<0.001	0.001	0.014	4.08
Biological–demographic	31.34 (2)	<0.001	<0.001	<0.001	0.005	5.23
Four core paths [a]	54.14 (4)	<0.001	<0.001	<0.001	0.001	5.71

Δβ, difference between the physical and psychological coefficients (physical minus psychological), from the survey-weighted multiple-indicator multiple-cause model reported in the main text (Table 3) and in Table S15; SE, standard error; χ^2, Wald chi-square statistic; df, degrees of freedom. Each p-value is recomputed after multiplying the standard error of the coefficient difference by the square root of the assumed design effect; for the omnibus tests, the Wald χ^2 statistic is equivalently divided by the assumed design effect. This extends to the path-difference tests the same uniform-inflation assumption applied to the individual associations in Table S14, namely that unaccounted clustering inflates the coefficient covariance matrix by a common factor. A design effect of 1.0 corresponds to the analysis as reported. The critical design effect is the assumed value at which the test would cease to be significant at $\alpha = 0.05$; it is shown only for tests significant in the reported analysis (—, not significant at a design effect of 1.0). Rows in bold are the four path differences that were significant in the primary model and in every sensitivity specification (Table S15). [a] Peer victimization, parental support, sex, and chronic illness tested jointly.

Section S7: Network Structure of Somatic Dimensions and Their Correlates

The ordinal regression models in Section S5 quantify how each predictor relates to physical and psychological somatic burden one variable at a time, but they do not represent the system as a whole: they cannot show how the predictors relate to one another, nor whether the two somatic dimensions occupy distinct positions within a wider web of associations (Haslbeck & Waldorp, 2020). To recover this system-level structure, we estimated a mixed graphical model (MGM), in which each variable is a node and each edge is a regularized partial association between two nodes conditional on all others. This representation complements the regression analysis: rather than asking how strongly each predictor predicts somatic burden, it asks how the somatic dimensions are embedded among their correlates, which variables are central, and whether physical and psychological burden connect to different parts of the system.

Model specification and estimation

The network comprised fifteen nodes: the two continuous somatic burden scores (physical and psychological), the three psychosocial variables (bullying exposure, school alienation, and parental support), the six lifestyle and socioeconomic indicators (BMI z-score, healthy diet, unhealthy diet, physical activity, income strain, and housing problems), age, and the three binary or categorical variables (sex, chronic illness, and organized sport). The MGM accommodates this mixed set of variable types directly: continuous nodes were modeled as conditionally Gaussian and the binary nodes as categorical, so that no node was forced onto a distributional scale inconsistent with its measurement. Edge weights were estimated by nodewise l_1-regularized regression, with the regularization parameter selected by the extended Bayesian information criterion ($\gamma = 0.25$) and edges retained under the AND rule, so that an edge appears only when both nodewise regressions identify it. The model was estimated on the unweighted analytic sample ($n = 3523$). As with the confirmatory factor, correspondence, and latent profile analyses, the network was estimated without survey weights because it characterizes conditional dependence structure rather than population-level prevalence; weighting affects the generalization of point estimates rather than the recovery of structure. Continuous nodes were standardized prior to estimation. Node predictability, the proportion of variance (continuous nodes, R^2) or the normalized accuracy beyond the marginal (categorical nodes, normalized correct classification) explained by each node's neighbors, was computed to quantify how much of each node is accounted for by the rest of the network.

Edge structure and dimensional separation

The estimated network was sparse, retaining 23 of 105 possible edges, of which 20 had 95% bootstrap confidence intervals that excluded zero (see below). The strongest edge by a wide margin connected the two somatic dimensions themselves (physical–psychological, weight 0.49), indicating a substantial shared somatic core that persists after conditioning on every measured correlate. Beyond this core, the two dimensions connected to distinct regions of the system, mirroring the dissociation observed in the ordinal models. Physical somatic burden was most strongly tied to the biological and demographic nodes: sex (0.20) and chronic illness (0.11). Psychological somatic burden was instead most strongly tied to the psychosocial nodes: bullying exposure (0.16), school alienation (0.09), and, negatively, parental support (−0.045). Bullying exposure and school alienation formed their own tight cluster (0.21), and parental support connected negatively to school alienation (−0.19), recovering the internal coherence of the psychosocial microsystem. A separate cluster of expected, largely mechanical associations linked the lifestyle and socioeconomic nodes (physical activity–organized sport, 0.35; income strain–housing problems, 0.24), confirming that the network reproduced known structure while leaving the somatic dimensions to connect to their substantive correlates. The full set of bootstrap-validated edges involving the somatic dimensions is reported in Table S17.

Table S17. Bootstrap-validated network edges involving the somatic dimensions, grouped by neighbor system

Neighbor node	Partial association	95% CI	Direction	System
Somatic core				
Physical – Psychological somatic	0.49	0.46, 0.52	Positive	Somatic
Physical somatic burden correlates				
Sex	0.20	0.15, 0.25	Female > male	Demographic
Chronic illness	0.11	0.01, 0.20	Present > absent	Biological
Bullying exposure	0.06	0.01, 0.12	Positive	Psychosocial
Age	0.05	0.00, 0.10	Positive	Demographic
Psychological somatic burden correlates				
Bullying exposure	0.16	0.11, 0.20	Positive	Psychosocial
Organized sport	0.10	0.03, 0.18	Participates > not	Lifestyle
School alienation	0.09	0.06, 0.13	Positive	Psychosocial
Age	0.05	0.01, 0.09	Positive	Demographic
Parental support	−0.04	−0.09, −0.00	Negative	Psychosocial

Edges are from a mixed graphical model estimated by EBIC-selected nodewise l_1-regularized regression ($\gamma = 0.25$, AND rule) on the unweighted analytic sample (n = 3523), grouped by which somatic dimension they attach to and by the system of the neighbor node. Partial associations are conditional on all other nodes. Confidence intervals are 95% nonparametric bootstrap intervals (1000 resamples); only edges whose intervals exclude zero are listed. For edges between a continuous and a categorical node (sex, chronic illness, organized sport), the partial association is symmetric and unsigned at the edge level; the direction column reports the sign of the difference in the continuous neighbor between the reference and comparison levels (female, chronic illness present, and sport participation, respectively), derived from the nodewise coefficients. The grouping makes explicit the dissociation described in the main text: physical somatic burden attaches preferentially to biological and demographic neighbors, whereas psychological somatic burden attaches preferentially to psychosocial neighbors. Values below 0.005 in magnitude are shown as 0.00 (Table S17).

Centrality and predictability

Node strength centrality, the sum of the absolute edge weights attached to each node, placed the two somatic dimensions among the most central nodes in the network (psychological somatic and physical somatic ranked first and third), consistent with their role as the substantive hubs around which the system is organized; organized sport ranked second, reflecting its dense ties within the lifestyle cluster. The predictability of the somatic nodes was modest: the network accounted for 34% of the variance in physical somatic burden and 37% in psychological somatic burden ($R^2 = 0.34$ and 0.37). Because a large share of this explained variance is carried by the strong physical–psychological edge rather than by the external correlates, the predictability attributable to the measured psychosocial, lifestyle, and demographic nodes individually is small. This ceiling closely matches the modest classification performance of the machine-learning models reported in Section S8, and reinforces the interpretation that a substantial portion of adolescent somatic burden lies beyond the variables measured here.

Edge stability

The accuracy and stability of the network were assessed with the nonparametric and case-dropping bootstrap (bootnet; 1000 resamples each) (Epskamp et al., 2018). The edge-weight bootstrap confidence intervals were narrow for the strong edges and excluded zero for 20 of the 23 nonzero edges, including every edge involving the somatic dimensions reported in Table S17. The case-dropping bootstrap indicated that strength centrality was highly stable: the correlation-stability coefficient reached its maximum tested value (CS-coefficient = 0.75), meaning that the centrality ordering was preserved in 95% of samples even when 75% of cases were dropped, well above the 0.50 threshold recommended for interpreting centrality with confidence. The network structure summarized in the main text (Figure 3) is therefore robust, notwithstanding the modest absolute magnitude of the external edges that follows from the weak association common to all analyses in this study.

The network and the regression are described in the main text as convergent, and their substantive conclusions do converge. They do not, however, answer the same question, and one apparent inconsistency between them requires explanation. In the regression, participation in organized sport is associated with higher burden on both dimensions, with odds ratios of 1.42 and 1.57. In the mixed graphical model, organized sport is connected to psychological burden but has no edge to physical burden.

This is a consequence of conditioning rather than a contradiction. Each regression estimates the association between a predictor and one somatic dimension without conditioning on the other. The network conditions every node on all remaining nodes, including the other somatic dimension, with which it shares the strongest edge in the graph. Any association that a predictor has with one dimension only by way of the core that the two dimensions share is therefore absorbed once that core is held constant.

Table S18. Conditional models reconciling the regression and the network

Panel A. Odds ratios before and after conditioning on the other somatic dimension

Predictor	Physical, unconditional	Physical, conditional	Psychological, unconditional	Psychological, conditional
**Peer victimization**	**1.429**	**1.125**	**1.840**	**1.596**
**School alienation**	**1.121**	**1.009**	**1.177**	**1.119**
Parental support	1.001	1.104	0.883	0.884
BMI z-score	0.958	0.961	0.988	1.005
Healthy diet	0.842	0.878	0.860	0.916
Unhealthy diet	1.004	0.983	1.041	1.041
Physical activity	1.061	1.022	1.114	1.115
**Chronic illness**	**1.612**	**1.499**	**1.374**	**1.024**
**Organized sport**	**1.421**	**1.179**	**1.573**	**1.440**
Income strain	0.988	0.942	1.044	1.043
Housing problems	1.054	1.069	0.968	0.931
Age	1.259	1.152	1.205	1.097
Sex (female)	2.351	1.880	1.820	1.215
Other somatic dimension	—	*3.14*	—	*3.44*

Panel B. Nodewise partial coefficients, estimate (p)

Predictor	Physical node, complete case	Physical node, imputed	Psychological node, complete case	Psychological node, imputed
**Peer victimization**	**0.078 (<0.001)**	**0.080 (<0.001)**	**0.150 (<0.001)**	**0.158 (<0.001)**
**School alienation**	**0.001 (0.974)**	**0.000 (0.981)**	**0.093 (<0.001)**	**0.094 (<0.001)**
Parental support	0.031 (0.032)	0.023 (0.082)	−0.073 (<0.001)	−0.067 (<0.001)
BMI z-score	−0.010 (0.448)	0.004 (0.743)	0.005 (0.731)	−0.006 (0.628)
Healthy diet	−0.046 (0.001)	−0.046 (<0.001)	−0.022 (0.104)	−0.017 (0.196)
Unhealthy diet	0.007 (0.635)	0.002 (0.903)	0.004 (0.790)	0.013 (0.302)
Physical activity	−0.017 (0.225)	−0.007 (0.577)	0.039 (0.005)	0.028 (0.030)
**Chronic illness**	**0.205 (<0.001)**	**0.193 (<0.001)**	**0.017 (0.694)**	**0.046 (0.265)**
**Organized sport**	**0.025 (0.476)**	**0.012 (0.711)**	**0.173 (<0.001)**	**0.178 (<0.001)**
Income strain	−0.003 (0.834)	0.006 (0.661)	0.029 (0.042)	0.024 (0.071)
Housing problems	0.036 (0.011)	0.027 (0.044)	−0.025 (0.074)	−0.017 (0.189)
Age	0.060 (<0.001)	0.064 (<0.001)	0.060 (<0.001)	0.059 (<0.001)
Sex (female)	0.274 (<0.001)	0.270 (<0.001)	0.109 (<0.001)	0.096 (<0.001)
Other somatic dimension	*0.509 (<0.001)*	*0.503 (<0.001)*	*0.492 (<0.001)*	*0.485 (<0.001)*

Panel A refits each survey-weighted ordinal model with the standardized score of the other somatic dimension entered as an additional covariate. Panel B reports unregularized nodewise regressions, which correspond to the network without its penalty, in the complete-case sample and pooled across 30 imputed datasets. Rows in bold are the predictors whose unique association is confined to one dimension.

The two panels agree. Once the shared somatic core is held constant, organized sport retains a clear association with psychological burden but none whatever with physical burden. The same logic operates in the opposite direction: chronic illness retains its association with physical burden but loses its association with psychological burden, and school alienation retains its association with psychological burden but has no unique association with physical burden. Peer victimization is the one exposure that retains an independent association with both dimensions, though it remains the stronger for psychological burden.

The absence of a sport-to-physical edge in the network is therefore the expected consequence of conditioning rather than a discrepancy between methods, and the unique associations recovered by the network are more sharply dissociated than the marginal associations recovered by the regression. The two approaches agree; they differ in what they hold constant, and this difference is informative rather than troubling.

Section S8: Machine-Learning Validation of Predictor Importance

As a complement to the survey-weighted ordinal regression that does not impose the proportional-odds functional form, we evaluated whether flexible machine-learning algorithms (a) recover the same ranking of important predictors and (b) detect any nonlinearity or interaction structure that the additive proportional-odds models might miss. The machine-learning analysis is complementary rather than primary: the ordinal regression provides the principal inferential results, and the role of the algorithms here is to test the robustness of the variable-importance pattern under models that do not impose that functional form.

Design and algorithms

Each somatic outcome was modeled in its three-category form (low, moderate, high), parallel to the ordinal analysis, with physical and psychological burden treated as two separate classification problems. The three-class categorical form was preferred over the continuous scores because the latter were right-skewed and zero-inflated (31.8% and 27.7% zeros for physical and psychological burden; skewness 1.39 and 0.99); the resulting classes were approximately balanced, so no imbalanced-learning correction was required. Three algorithms spanning distinct modeling philosophies were fitted: an interpretable penalized-regression baseline (elastic net) and two tree-based ensembles suited to mixed-type predictors and nonlinearity (random forest and extreme gradient boosting). The elastic net was included not to address multicollinearity, which was absent in these data (all variance inflation factors below 1.2, Section S5), but to provide a linear, regularized reference point estimated and evaluated within the same cross-validation framework as the ensembles; its role is to establish the performance attainable by a purely additive linear model, so that any improvement by the tree-based ensembles can be read as evidence of nonlinearity or interaction structure, and the absence of such improvement as evidence that the additive structure is sufficient. The thirteen predictors were the same as in the ordinal models. Models were trained on a stratified 70% partition (n = 2468) and evaluated on the held-out 30% test set (n = 1055), with all hyperparameters tuned by 10-fold cross-validation within the training partition under an identical resampling scheme across algorithms. Nested cross-validation was unnecessary given the large ratio of observations to predictors (271 observations per predictor). As with the network analysis, the models were fitted unweighted, consistent with their structural role and with their use as a complementary robustness and triangulation analysis rather than as a confirmatory test. A fixed random seed governed all partitioning and tuning to ensure reproducibility.

Classification performance

The three algorithms achieved closely comparable and modest discrimination on the held-out test set, with multiclass area under the curve (AUC) clustered between 0.64 and 0.66 for both outcomes (Table S19). No algorithm meaningfully outperformed the others: the penalized-regression baseline, the bagging ensemble, and the boosting ensemble converged on the same performance ceiling, and the tuned tree-based models repeatedly selected conservative configurations (shallow trees, large minimum node sizes), indicating little exploitable nonlinearity or interaction beyond the additive structure already captured by the regression. That three distinct learning paradigms reach the same ceiling indicates that the limit is a property of the data rather than of any one model: the measured predictors carry only a modest amount of information about somatic burden, consistent with the network predictability of 0.34 and 0.37 reported in Section S7. This modest performance is itself an informative, honest finding for a system-level account in which a substantial proportion of the variance in somatic burden remains unexplained by the measured predictors.

Table S19. Cross-validated and held-out classification performance of the three algorithms

Outcome	Algorithm	AUC	Accuracy	Cohen's κ
Physical somatic burden	Elastic net	0.635	0.457	0.166
	Random forest	0.640	0.474	0.192
	XGBoost	0.647	0.461	0.176
Psychological somatic burden	Elastic net	0.646	0.441	0.123
	Random forest	0.657	0.454	0.151
	XGBoost	0.654	0.451	0.153

Performance is reported on the held-out test set (n = 1055). AUC, multiclass area under the receiver operating characteristic curve (one-vs-rest); Accuracy and Cohen's κ summarize three-class agreement. Hyperparameters were tuned by 10-fold cross-validation within the training partition (n = 2468) under an identical scheme across algorithms; models were fitted unweighted. XGBoost was fitted through its native interface with a manually implemented 10-fold cross-validation using identical folds and selection metric, owing to an incompatibility between recent xgboost releases and the cross-validation wrapper.

Variable importance and SHAP analysis

Permutation importance from the random forest and SHAP (Shapley additive explanations) values from the gradient-boosting model converged on the same ordering, which in turn reproduced the dissociation found in the ordinal regression and the network. SHAP values were computed on the held-out test set for the probability of the high category, so that the explanation reflects out-of-sample behavior. For physical somatic burden, the most important predictors were bullying exposure and sex, followed by age, whereas for psychological somatic burden, bullying exposure and school alienation dominated, with sex falling to fourth (Table S20). The direction of every leading effect matched the regression: female sex and chronic illness raised the probability of high physical burden, higher parental support lowered the probability of high psychological burden, and greater bullying exposure raised both. The beeswarm summary of the SHAP values for the best-performing model is shown in Figure S2. The convergence of four methodologically distinct approaches, survey-weighted ordinal regression, a mixed graphical model, permutation importance, and SHAP, on the same dissociation, psychosocial systems aligning with psychological burden and biological and demographic factors with physical burden, provides convergent robustness evidence for the central finding.

Table S20. Predictor importance for the high somatic category (mean absolute SHAP)

Rank	Physical somatic burden	Psychological somatic burden
1	Bullying exposure (0.229)	Bullying exposure (0.244)
2	Sex – female (0.215)	School alienation (0.206)
3	Age (0.138)	Parental support (0.139)
4	Parental support (0.097)	Sex – female (0.126)
5	Healthy diet (0.086)	Age (0.091)
6	Chronic illness (0.069)	Healthy diet (0.071)
7	Organized sport (0.057)	Organized sport (0.059)
8	School alienation (0.047)	Physical activity (0.037)

Values are the mean absolute SHAP value for each predictor, computed on the held-out test set (n = 1055) for the probability of the high somatic category from the gradient-boosting model; higher values indicate greater contribution. The eight highest-ranked predictors are shown for each outcome. The ranking reproduces the dissociation observed in the ordinal regression and the network (Section S7): psychosocial variables rank highest for psychological burden, whereas sex and chronic illness rank higher for physical burden (Table S12).

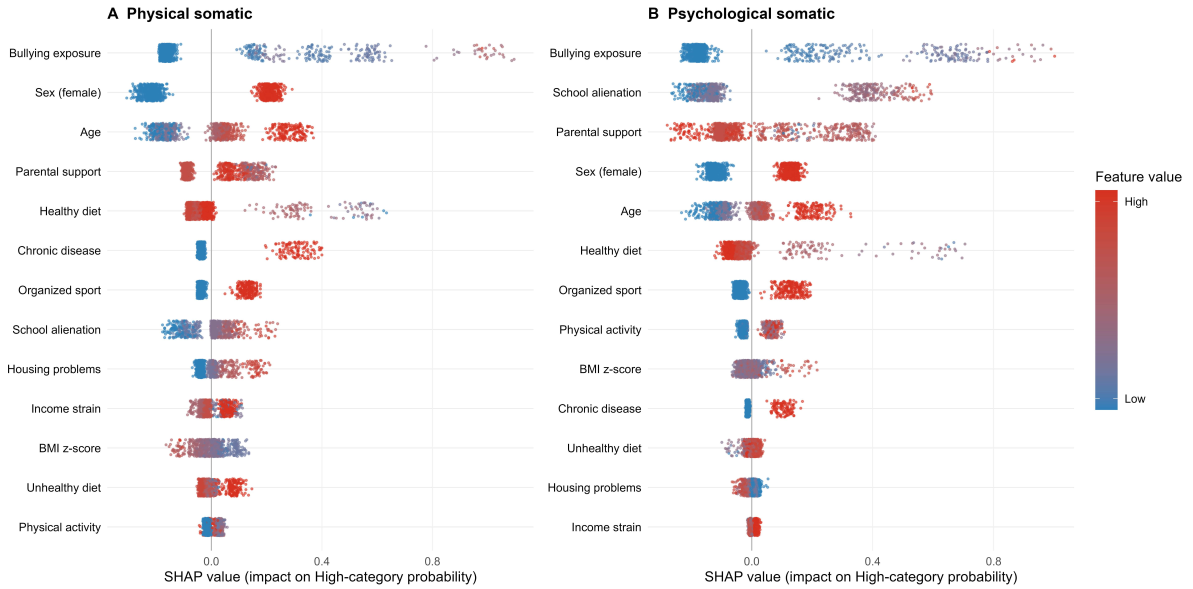

Figure S2. SHAP summary for the gradient-boosting model

In Figure S2, each point represents a participant in the held-out test set. The horizontal axis shows the SHAP value, that is, the contribution of a given predictor to the model's estimated probability of membership in the high somatic category. Point color reflects the observed predictor value, ranging from low values in blue to high values in red. Predictors are ranked by their mean absolute SHAP value, with the most influential predictors appearing at the top. Panel A presents the model for physical somatic burden, whereas Panel B presents the model for psychological somatic burden. Positive SHAP values indicate that the predictor increases the estimated probability of being in the high category. The different predictor patterns across the two panels—sex being more prominent for physical burden, and psychosocial predictors being more prominent for psychological burden—are consistent with the ordinal regression and network results.

The beeswarm display in Figure S2 conveys not only which predictors matter but how their effect is distributed across adolescents and in which direction. Several features are worth noting. First, the color gradient confirms the direction of each leading effect. For physical somatic burden (panel A), the sex point cloud separates cleanly into two blocks, with high-value (female) cases carrying positive SHAP values and low-value (male) cases negative, the sharpest directional separation of any predictor in either panel; chronic illness shows the same pattern, with the small group of affected adolescents forming a distinct positive cluster. Parental support appears in red on the negative side of zero in both panels, visually encoding its inverse contribution: higher support pushes the estimate away from the high category. Second, the spread of each predictor reveals heterogeneity of effect. Bullying exposure ranks first in both panels not because it shifts every adolescent, but because of a long right tail: the large majority cluster near zero (most adolescents report no victimization), while a minority with high exposure carry very large positive SHAP values, exactly the signature of a strongly skewed predictor with a concentrated, high-impact subgroup. Third, the panels make the central dissociation directly visible: sex is the second most influential predictor for physical burden but recedes for psychological burden, where school alienation and parental support rise to prominence, so that the psychosocial predictors visibly dominate the upper rows of panel B while the biological and demographic predictors dominate panel A. Fourth, the predictors that were negligible in the regression, BMI z-score, unhealthy diet, income strain, and housing problems, form tight, near-zero point clouds in both panels, confirming their limited contribution under a model that makes no linearity assumption.

Section S9: Statistical Software and Reproducibility

This study used data from the 2022 Türkiye Child Survey conducted by the Turkish Statistical Institute (TURKSTAT). The microdata are not publicly available owing to confidentiality restrictions imposed by TURKSTAT. Researchers wishing to access the data may submit a formal application through TURKSTAT's official data access procedures.

All statistical analyses were performed using R version 4.5.2. Data management, statistical modeling, and visualization were conducted using the packages listed in Table S21.

Table S21. R packages used for statistical analysis

Package	Version	Purpose
bootnet (Epskamp et al., 2018)	1.8	Network accuracy and stability (bootstrap)
car (Fox & Weisberg, 2024)	3.1-5	Variance inflation factors
caret (Kuhn, 2008)	7.0-1	Model training, tuning, and resampling
coin (Hothorn et al., 2008)	1.4-3	Linear-by-linear association (ordinal trend) tests
dplyr (Wickham et al., 2023)	1.1.4	Data manipulation and transformation
factoextra (Kassambara & Mundt, 2026)	2.0.0	Extraction and visualization of MCA results
FactoMineR (Lê et al., 2008)	2.14	Multiple correspondence analysis
finalfit (Harrison et al., 2025)	1.1.0	Included-versus-excluded comparison tables
ggnewscale (Campitelli, 2025)	0.5.2	Multiple color and fill scales
ggplot2 (Wickham, 2016)	4.0.3	Construction of the study figures
ggrepel (Slowikowski, 2026)	0.9.8	Non-overlapping text labels
glmnet (Tay et al., 2023)	5.0	Elastic-net penalized regression
lavaan (Rosseel, 2012; Rosseel et al., 2025)	0.6-21	Confirmatory factor analysis (WLSMV) and the multiple-indicator multiple-cause model with Wald tests of equality constraints
MASS (Venables & Ripley, 2002)	7.3-65	Support for ordinal logistic regression
mgcv (Wood, 2017)	1.9-4	Generalized additive models (nonlinearity checks)
mgm (Haslbeck & Waldorp, 2020)	1.2-15	Mixed graphical model estimation
mice (van Buuren & Groothuis-Oudshoorn, 2011)	3.19.0	Multiple imputation by predictive mean matching
MLmetrics (Yan, 2024)	1.1.3	Classification performance metrics
naniar (Tierney & Cook, 2023)	1.1.0	Missing-data summaries and Little's MCAR test
patchwork (Pedersen, 2025)	1.3.2	Multi-panel figure composition
pROC (Robin et al., 2011)	1.19.0.1	Multiclass ROC curves and area under the curve
psych (Revelle, 2025)	2.6.5	Polychoric correlations and ordinal reliability
qgraph (Epskamp et al., 2012)	1.9.8	Network estimation and visualization
ranger (Wright & Ziegler, 2017)	0.18.0	Random forest with permutation importance
readxl (Wickham & Bryan, 2026)	1.5.0	Importing the source microdata
reshape2 (Wickham, 2007)	1.4.5	Reshaping data for the correlation-matrix figure
semTools (Jorgensen et al., 2022)	0.5-8	Heterotrait-monotrait (HTMT) ratio
stringr (Wickham, 2025)	1.6.0	String operations in variable preparation
survey (Lumley, 2024)	4.5	Survey design objects and design-based inference
tidyLPA (Rosenberg et al., 2018)	2.0.2	Latent profile analysis and model enumeration
xgboost (T. Chen et al., 2026)	3.2.1.1	Extreme gradient boosting and SHAP contributions

A fixed random seed (set.seed(2026)) was specified before the training–test partition and the cross-validation and bootstrap routines, and a separate seed before the latent profile enumeration, to ensure reproducibility. The analysis code and aggregate outputs are openly available on GitHub (https://github.com/sy142/adolescent-somatic-systems) and permanently archived on Zenodo (https://doi.org/10.5281/zenodo.20631358).

References for Supplementary Material

Campitelli, E. (2025). *ggnewscale: Multiple Fill and Colour Scales in "ggplot2."* https://doi.org/10.32614/CRAN.package.ggnewscale

Chen, S., Zhang, H., Gao, M., Machado, D. B., Jin, H., Scherer, N., Sun, W., Sha, F., Smythe, T., Ford, T. J., & Kuper, H. (2024). Dose-Dependent Association Between Body Mass Index and Mental Health and Changes Over Time. *JAMA Psychiatry*, *81*(8), 797. https://doi.org/10.1001/jamapsychiatry.2024.0921

Chen, T., He, T., Benesty, M., Khotilovich, V., Tang, Y., Cho, H., Chen, K., Mitchell, R., Cano, I., Zhou, T., Li, M., Xie, J., Lin, M., Geng, Y., Li, Y., Yuan, J., & Cortes, D. (2026). *xgboost: Extreme Gradient Boosting*. https://doi.org/10.32614/CRAN.package.xgboost

Dunn, K. J., & McCray, G. (2020). The Place of the Bifactor Model in Confirmatory Factor Analysis Investigations Into Construct Dimensionality in Language Testing. *Frontiers in Psychology*, *11*. https://doi.org/10.3389/fpsyg.2020.01357

Epskamp, S., Borsboom, D., & Fried, E. I. (2018). Estimating psychological networks and their accuracy: A tutorial paper. *Behavior Research Methods*, *50*(1), 195–212. https://doi.org/10.3758/s13428-017-0862-1

Epskamp, S., Cramer, A. O. J., Waldorp, L. J., Schmitt, V. D., & Borsboom, D. (2012). qgraph: Network Visualizations of Relationships in Psychometric Data. *Journal of Statistical Software*, *48*(4), 1–18.

Fornell, C., & Larcker, D. F. (1981). Evaluating Structural Equation Models with Unobservable Variables and Measurement Error. *Journal of Marketing Research*, *18*(1), 39–50. https://doi.org/10.1177/002224378101800104

Fox, J., & Weisberg, S. (2024). *car: Companion to Applied Regression (Version 3.1-5) [R package]. Comprehensive R Archive Network (CRAN). https://CRAN.R-project.org/package=car (Accessed June 10, 2026)*.

Harrell, F. E. (2015). *Regression Modeling Strategies*. Springer International Publishing. https://doi.org/10.1007/978-3-319-19425-7

Harrison, E., Drake, T., & Pius, R. (2025). *finalfit: Quickly Create Elegant Regression Results Tables and Plots when Modelling*. https://doi.org/10.32614/CRAN.package.finalfit

Haslbeck, J. M. B., & Waldorp, L. J. (2020). mgm: Estimating Time-Varying Mixed Graphical Models in High-Dimensional Data. *Journal of Statistical Software*, *93*(8). https://doi.org/10.18637/jss.v093.i08

Hothorn, T., Hornik, K., Wiel, M. A. van de, & Zeileis, A. (2008). Implementing a Class of Permutation Tests: The coin Package. *Journal of Statistical Software*, *28*(8). https://doi.org/10.18637/jss.v028.i08

Jöreskog, K. G., & Goldberger, A. S. (1975). Estimation of a Model with Multiple Indicators and Multiple Causes of a Single Latent Variable. *Journal of the American Statistical Association*, *70*(351), 631–639. https://doi.org/10.1080/01621459.1975.10482485

Jorgensen, T. D., Pornprasertmanit, S., Schoemann, A. M., & Rosseel, Y. (2022). *semTools: Useful tools for structural equation modeling* (0.5-6). CRAN.

Kassambara, A., & Mundt, F. (2026). *factoextra: Extract and Visualize the Results of Multivariate Data Analyses*. https://CRAN.R-project.org/package=factoextra

Kenny, D. A., Kaniskan, B., & McCoach, D. B. (2015). The Performance of RMSEA in Models With Small Degrees of Freedom. *Sociological Methods & Research*, *44*(3), 486–507. https://doi.org/10.1177/0049124114543236

Kish, L. (1965). *Survey Sampling* (1st ed.). John Wiley & Sons.

Kuhn, M. (2008). Building Predictive Models in R Using the caret Package. *Journal of Statistical Software*, *28*(5). https://doi.org/10.18637/jss.v028.i05

Lê, S., Josse, J., & Husson, F. (2008). FactoMineR : An R Package for Multivariate Analysis. *Journal of Statistical Software*, *25*(1). https://doi.org/10.18637/jss.v025.i01

Little, R. J. A. (1988). A Test of Missing Completely at Random for Multivariate Data with Missing Values. *Journal of the American Statistical Association*, *83*(404), 1198–1202. https://doi.org/10.1080/01621459.1988.10478722

Lumley, T. (2024). *survey: analysis of complex survey samples* (4.4). CRAN R-project.

Nylund-Gibson, K., & Choi, A. Y. (2018). Ten frequently asked questions about latent class analysis. *Translational Issues in Psychological Science*, *4*(4), 440–461. https://doi.org/10.1037/tps0000176

Pedersen, T. L. (2025). *patchwork: The Composer of Plots*. https://doi.org/10.32614/CRAN.package.patchwork

Reise, S. P., Bonifay, W. E., & Haviland, M. G. (2013). Scoring and Modeling Psychological Measures in the Presence of Multidimensionality. *Journal of Personality Assessment*, *95*(2), 129–140. https://doi.org/10.1080/00223891.2012.725437

Revelle, W. (2025). *psych: Procedures for Psychological, Psychometric, and Personality Research* (2.5.6). CRAN R-project.

Robin, X., Turck, N., Hainard, A., Tiberti, N., Lisacek, F., Sanchez, J.-C., & Müller, M. (2011). pROC: an open-source package for R and S+ to analyze and compare ROC curves. *BMC Bioinformatics*, *12*(1), 77. https://doi.org/10.1186/1471-2105-12-77

Rodriguez, A., Reise, S. P., & Haviland, M. G. (2016). Evaluating bifactor models: Calculating and interpreting statistical indices. *Psychological Methods*, *21*(2), 137–150. https://doi.org/10.1037/met0000045

Rosenberg, J., Beymer, P., Anderson, D., van Lissa, C. j., & Schmidt, J. (2018). tidyLPA: An R Package to Easily Carry Out Latent Profile Analysis (LPA) Using Open-Source or Commercial Software. *Journal of Open Source Software*, *3*(30), 978. https://doi.org/10.21105/joss.00978

Rosseel, Y. (2012). lavaan: An R Package for Structural Equation Modeling. *Journal of Statistical Software*, *48*(2), 1–36. https://doi.org/10.18637/jss.v048.i02

Rosseel, Y., Jorgensen, T. D., & De Wilde, L. (2025). *lavaan: Latent Variable Analysis* (0.6-21.tar.gz). https://doi.org/10.32614/CRAN.package.lavaan

Rubin, D. B. (1987). *Multiple Imputation for Nonresponse in Surveys*. Wiley. https://doi.org/10.1002/9780470316696

Slowikowski, K. (2026). *ggrepel: Automatically Position Non-Overlapping Text Labels with "ggplot2."* https://doi.org/10.32614/CRAN.package.ggrepel

Spurk, D., Hirschi, A., Wang, M., Valero, D., & Kauffeld, S. (2020). Latent profile analysis: A review and "how to" guide of its application within vocational behavior research. *Journal of Vocational Behavior*, *120*, 103445. https://doi.org/10.1016/j.jvb.2020.103445

Tay, J. K., Narasimhan, B., & Hastie, T. (2023). Elastic Net Regularization Paths for All Generalized Linear Models. *Journal of Statistical Software*, *106*(1). https://doi.org/10.18637/jss.v106.i01

Tierney, N., & Cook, D. (2023). Expanding Tidy Data Principles to Facilitate Missing Data Exploration, Visualization and Assessment of Imputations. *Journal of Statistical Software*, *105*(7), 1–31. https://doi.org/10.18637/jss.v105.i07

Turkish Statistical Institute. (2023). *Türkiye Child Survey, 2022*.

van Buuren, S., & Groothuis-Oudshoorn, K. (2011). mice: Multivariate Imputation by Chained Equations in R. *Journal of Statistical Software*, *45*(3), 1–67. https://doi.org/10.18637/jss.v045.i03

Venables, W. N., & Ripley, B. D. (2002). *Modern Applied Statistics with S* (MASS package in R). Springer New York. https://doi.org/10.1007/978-0-387-21706-2

Wickham, H. (2007). Reshaping Data with the reshape Package. *Journal of Statistical Software*, *21*(12). https://doi.org/10.18637/jss.v021.i12

Wickham, H. (2016). ggplot2: Elegant Graphics for Data Analysis. In *Elegant Graphics for Data Analysis* (2nd ed., ggplot2. Use R!). Springer. https://doi.org/10.1007/978-3-319-24277-4

Wickham, H. (2025). *stringr: Simple, Consistent Wrappers for Common String Operations*. https://doi.org/10.32614/CRAN.package.stringr

Wickham, H., Francois, R., Henry, L., Muller, K., & Vaughan, D. (2023). *dplyr: A Grammar of Data Manipulation. R package version 1.1.4* (R package version 1.1.4). CRAN R-project.

Wood, S. N. (2017). *Generalized additive models: an introduction with R*. CRC Press/Taylor & Francis Group.

Wright, M. N., & Ziegler, A. (2017). ranger : A Fast Implementation of Random Forests for High Dimensional Data in C++ and R. *Journal of Statistical Software*, *77*(1). https://doi.org/10.18637/jss.v077.i01

Yan, Y. (2024). *MLmetrics: Machine Learning Evaluation Metrics*. https://doi.org/10.32614/CRAN.package.MLmetrics
